# Efficacy and safety of oral sodium bicarbonate in kidney-transplant recipients and non-transplant patients with chronic kidney disease: a systematic review and meta-analysis

**DOI:** 10.3389/fphar.2024.1411933

**Published:** 2024-08-26

**Authors:** Yun Wu, Ying Wang, Weijun Huang, Xi Guo, Baoluo Hou, Jingyi Tang, Yuqi Wu, Huijuan Zheng, Yanling Pan, Wei Jing Liu

**Affiliations:** ^1^ Renal Research Institution of Beijing University of Chinese Medicine, and Key Laboratory of Chinese Internal Medicine of Ministry of Education and Beijing, Dongzhimen Hospital, Beijing University of Chinese Medicine, Beijing, China; ^2^ Department of Chinese Medicine, the Affiliated Hospital of Guizhou Medical University, Guiyang, China

**Keywords:** chronic kidney disease, kidney-transplant recipient, oral sodium bicarbonate, metabolic acidosis, patient, randomized controlled trial

## Abstract

**Introduction:**

We investigated the efficacy and safety of oral sodium bicarbonate in kidney-transplant recipients and non-transplant patients with chronic kidney disease (CKD), which are currently unclear.

**Methods:**

PubMed, Cochrane Library, Embase, and Web of Science were searched for randomized controlled trials investigating the efficacy and safety of sodium bicarbonate *versus* placebo or standard treatment in kidney-transplant and non-transplant patients with CKD.

**Results:**

Sixteen studies of kidney-transplant recipients (two studies, 280 patients) and non-transplant patients with CKD (14 studies, 1,380 patients) were included. With non-transplant patients, sodium bicarbonate slowed kidney-function declines (standardized mean difference [SMD]: 0.49, 95% confidence interval [CI]: 0.14–0.85, *p* = 0.006) within ≥12 months (SMD: 0.75 [95% CI: 0.12–1.38], *p* = 0.02), baseline-serum bicarbonate <22 mmol/L (SMD: 0.41 [95% CI: 0.19–0.64], *p* = 0.0004) and increased serum-bicarbonate levels (mean difference [MD]: 2.35 [95% CI: 1.40–3.30], *p* < 0.00001). In kidney-transplant recipients, sodium bicarbonate did not preserve graft function (SMD: -0.07 [95% CI: -0.30–0.16], *p* = 0.56) but increased blood pH levels (MD: 0.02 [95% CI: 0.00–0.04], *p* = 0.02). No significant adverse events occurred in the kidney-transplant or non-transplant patients (risk ratio [RR]: 0.89, [95% CI: 0.47–1.67], *p* = 0.72; and RR 1.30 [95% CI: 0.84–2.00], *p* = 0.24, respectively). However, oral sodium bicarbonate correlated with increased diastolic pressure and worsened hypertension and edema (MD: 2.21 [95% CI: 0.67–3.75], *p* = 0.005; RR: 1.44 [95% CI: 1.11–1.88], *p* = 0.007; and RR: 1.28 [95% CI: 1.00–1.63], *p* = 0.05, respectively).

**Discussion:**

Oral sodium bicarbonate may slow kidney-function decline in non-transplant patients with CKD taking sodium bicarbonate supplementation for ≥12 months or a baseline serum bicarbonate level of <22 mmol/L, without preserving graft function in kidney-transplant recipients. Sodium bicarbonate may increase diastolic pressure, and elevate a higher incidence of worsening hypertension and edema.

**Systematic Review Registration:**

https://www.crd.york.ac.uk/prospero/, identifier CRD42023413929.

## 1 Introduction

Chronic kidney disease (CKD), characterized by structural or functional abnormalities in the kidneys caused by various factors, affects approximately 9.1% of the global population (GBD Chronic Kidney Disease Collaboration, 2020), and its prevalence is on the rise (Xie et al., 2018). As the 10th leading cause of death worldwide (World Health Organization, 2023), CKD is an important public health issue. After patients with CKD progress to end-stage renal disease (ESRD), they require dialysis or kidney transplantation, which greatly increases medical expenses and social burdens (Guo et al., 2022; Garcia Sanchez et al., 2023; Bello et al., 2024), posing huge challenges to healthcare systems. Therefore, we focused on treating pre-ESRD (CKD stages G1–4) with the aim of discovering a therapeutic approach for delaying CKD progression. In patients with CKD after kidney transplantation, preserving the graft function to prevent them from returning to dialysis is a key concern.

Metabolic acidosis, a common complication of CKD, correlates with severe consequences, such as potential for CKD advancement (Madias, 2021), breakdown of skeletal muscles, resistance to insulin, loss of bone mineralization, and elevated mortality (Kovesdy, 2014; Garibotto et al., 2015; Bellasi et al., 2016; Raphael, 2019). Therefore, the 2024 Clinical Practice Guidelines of the Kidney Disease: Improving Global Outcomes (KDIGO) CKD Work Group (Kidney Disease: Improving Global Outcomes KDIGO CKD Work Group, 2024) recommend oral sodium bicarbonate for treating metabolic acidosis in patients with CKD. The results of multiple clinical trials have demonstrated that sodium bicarbonate corrects metabolic acidosis and delays the progression of kidney damage in patients with CKD who did not receive a transplant (Yan et al., 2017; Alva et al., 2020), even with normal serum bicarbonate concentrations (Mahajan et al., 2010), although other reports have shown that sodium bicarbonate did not provide this additional benefit (Bellasi et al., 2016; Kendrick et al., 2018; Bovée et al., 2021).

Metabolic acidosis is also highly prevalent in kidney-transplant recipients and is linked to graft function decline and increased mortality (Messa et al., 2016; Park et al., 2017; Gojowy et al., 2020). The incidence and severity of metabolic acidosis in kidney-transplant recipients are higher than in non-transplant individuals with CKD with similar kidney function (Messa et al., 2016). Metabolic acidosis has been identified as a significant risk factor for kidney-function deterioration in patients with CKD after kidney transplantation (Wiegand et al., 2022). A recent large randomized controlled trial (RCT) showed that sodium bicarbonate corrected metabolic acidosis but did not preserve graft function in kidney-transplant recipients (Mohebbi et al., 2023). However, no meta-analyses have been performed to provide a higher level of evidence. Whether sodium bicarbonate can delay the progression of kidney function in kidney-transplant recipients and non-transplant patients with CKD remains controversial.

In addition, sodium bicarbonate supplementation for metabolic acidosis in patients with CKD may cause several adverse events (AEs), the most concerning of which may be increased blood pressure, edema, and heart failure (Bushinsky, 2019; Raphael et al., 2020b). However, other findings have shown that sodium bicarbonate is not associated with adverse consequences (de Brito-Ashurst et al., 2009; Bellasi et al., 2016; Wiegand et al., 2022). Whether sodium bicarbonate supplementation is safe, especially in terms of blood pressure, for kidney-transplant recipients and non-transplant patients with CKD remains unclear. Therefore, in this study, we investigated the efficacy and safety of sodium bicarbonate in kidney-transplant recipients as well as non-transplant patients (stages G1–4), so as to provide stronger evidence for the clinical application of sodium bicarbonate.

## 2 Materials and methods

This study was conducted according to the guidelines of the Preferred Reporting Items for Systematic Reviews and Meta-Analyses (PRISMA) (Page et al., 2021) (Supplementary Table S1), and the protocol was recorded in the Prospective Register of Systematic Reviews under registration number CRD42023413929.

### 2.1 Search strategy

We comprehensively searched PubMed, the Cochrane Library, Embase, and the Web of Science for studies from inception until June 2023, using the keywords “renal insufficiency, chronic,” “sodium bicarbonate,” and “randomized controlled trial.” (Supplementary Table S2).

### 2.2 Inclusion and exclusion criteria

The inclusion criteria for this study were as follows: 1) the type of studies was limited to RCTs; 2) the participants were diagnosed with CKD (Kidney Disease: Improving Global Outcomes KDIGO CKD Work Group, 2024) stages G1–4 (estimated glomerular filtration rate [eGFR] ≥ 15 mL·min^–1^·1.73 m^–2^), including kidney-transplant recipients; 3) the included studies contained an intervention arm provided sodium bicarbonate (the dosage of sodium bicarbonate and duration of treatment were not restricted) and a control arm provided a placebo or standard treatment; 4) the included studies reported one or more of the following outcomes: changes in kidney function (eGFR or creatinine clearance [Ccl]), serum bicarbonate, blood pH, systolic blood pressure, diastolic blood pressure from baseline to the end of the study, or AEs (including edema, heart failure, worsening hypertension, and gastrointestinal disorders); and 5) the included studies were published in English. The presence of metabolic acidosis was not an inclusion criterion.

Studies were excluded if 1) the participants were diagnosed with ESRD (eGFR <15 mL·min^–1^·1.73 m^–2^), or dialysis-dependent patients, 2) patients were diagnosed with AKI, 3) the intervention arm or control arm was ineligible (for example, studies with the intervention arm provided intravenous sodium bicarbonate were excluded), 4) the complete text or data on outcome indicators were unavailable, 5) the studies were not published in English.

### 2.3 Study selection and data extraction

Two (Yun Wu and Ying Wang) of the authors independently screened and selected studies that met the inclusion criteria. If a trial included two or more groups with the same intervention, then the data from the same intervention were combined. If multiple secondary publications from the same trial were found, then the most comprehensive or recent dataset was used. Missing, unpublished, or incomplete data were obtained from the study investigators where possible. If the authors refused to provide data or we could not contact the authors, then the data from the study were excluded. Both authors independently used standardized tables for data extraction. The data extracted included the study characteristics (first author, year of publication, single or multicenter trial, intervention and control measures, dose of sodium bicarbonate, sample size, study duration, and trial quality as assessed using the Jadad score (Jadad et al., 1996)), the patient characteristics (mean age, male proportion, and baseline kidney function [eGFR or Ccl], the baseline serum-bicarbonate level, and the patient type), and reported outcomes (eGFR or Ccl, serum bicarbonate, blood pH, systolic blood pressure, diastolic blood pressure, and AEs, including edema, heart failure, worsening hypertension, and gastrointestinal disorders). Any differences of opinion were discussed with a third-party investigator (WJL) and settled by consensus. If the resulting data were represented by graphs, then the mean and standard deviation of the results were found and extracted from the graphs using the GetData software (version 2.25).

### 2.4 Quality assessment

Two authors (JYT and XG) independently assessed the risk of bias of the studies using the Cochrane Collaboration tool for randomized trials (Sterne et al., 2019) and documented the results using Review Manager (RevMan) software (version 5.4.1). The following items were assessed: random sequence generation, allocation concealment, blinding of participants and personnel, blinding of the outcome assessment, incomplete outcome data, selective reporting, and other biases. The Jadad score was used as a continuous variable to evaluate the quality of the included studies (Jadad et al., 1996). Discrepancies were settled by discussion and consensus with a third-party investigator (WJL). Additionally, the certainty of evidence across trials was assessed by using the Grading of Recommendations, Assessment, Development, and Evaluation (GRADE) approach (Guyatt et al., 2008).

### 2.5 Statistical analysis

Data analysis was conducted using RevMan software (version 5.4.1). For continuous outcomes, data were expressed as mean and SD. In the studies where SE values were initially reported, SD values were calculated following the Cochrane guidelines (Higgins et al., 2023). The mean difference in treatment effect from baseline to last measurement between treatment groups for each study was calculated, and the effect measure across all studies was expressed as the mean difference (MD) or standard mean difference (SMD) with a 95% confidence interval (CI). For dichotomous outcome data, the effect measure was expressed as a risk ratio (RR) with the 95% CI. Heterogeneity was evaluated with I^2^ statistics (where I^2^ < 50% and *p* > 0.10 indicated no significant heterogeneity), and a fixed-effect model was used. Otherwise, a random effect (RE) model was used.

According to the Cochrane Handbook (Higgins et al., 2023), if the heterogeneity of outcomes was high (I^2^ ≥ 50%) and >10 study comparisons were analyzed, we used Stata software (version 18.0) to carry out meta-regression analysis on the following variables: study duration, the Jadad score, the type of control group (placebo or standard treatment), year of publication, male ratio, the severity of kidney function at baseline and baseline mean serum-bicarbonate level to analyze the source of heterogeneity, with which univariate and multivariate analyses were performed. We concluded that heterogeneity and meta-regression were significant at *p* < 0.10 (the conservative criteria for meta-analysis). Then subgroup analyses were conducted according to the results of meta-regression to explore the source of heterogeneity further and calculate the pooled effects. Furthermore, to investigate whether the severity of metabolic acidosis affected the efficacy of sodium bicarbonate on eGFR or Ccl level, subgroup analysis was performed according to the baseline mean serum-bicarbonate level. To investigate whether kidney function affected the diastolic blood pressure and the incidence of edema induced by sodium bicarbonate, we conducted subgroup analysis according to the baseline mean eGFR or Ccl. The one-by-one elimination method (eliminating one study at a time and recalculating the results) was used for sensitivity analysis to evaluate the stability of the outcomes. Publication bias was assessed using Egger’s test to detect the asymmetry of the funnel plot if > 10 study comparisons were analyzed, with *p* > 0.05 indicating no publication bias (Higgins et al., 2023).

## 3 Results

### 3.1 Search results

**FIGURE 1:**
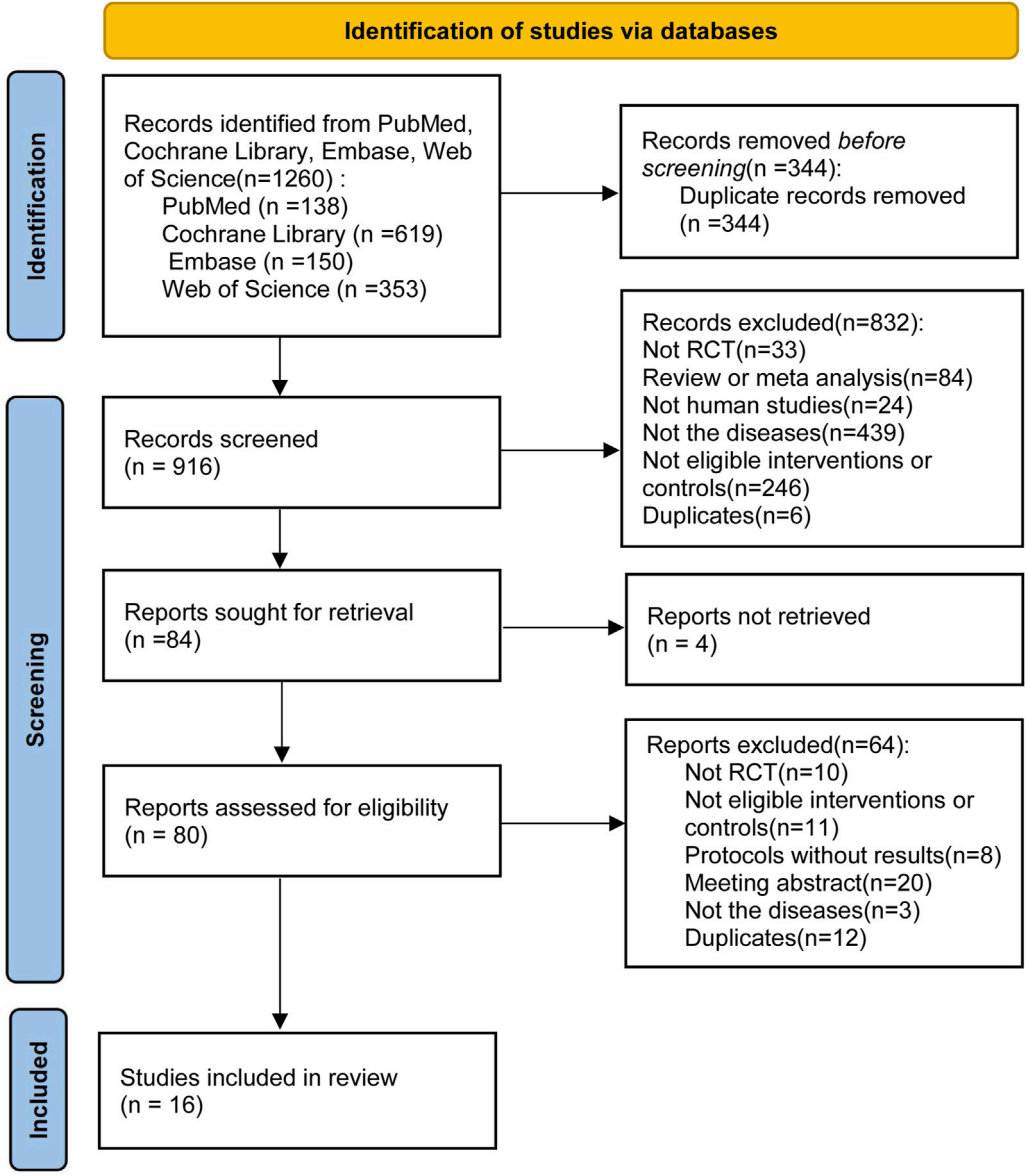
PRISMA flow chart describing the literature search and study selection.

Initially, we screened 1,260 articles by conducting literature searches of four electronic databases. After removing duplicate (n = 344) and unqualified manuscripts (n = 832) based on titles and abstracts, 84 potentially relevant articles remained for full-text review. Four articles were not retrieved, we excluded 64 articles for the following reasons: not RCT (n = 10); not eligible interventions or controls (n = 11); protocols without results (n = 8); conference abstract (n = 20); not the diseases (n = 3); duplicates (n = 12). A study (Aigner et al., 2019) reported the effect of sodium bicarbonate in patients with CKD and chronic metabolic acidosis but excluded for providing sodium bicarbonate in both treatment group and control group. Another study (Di Iorio et al., 2019) was excluded for including patients with CKD (stages G5, eGFR <15 mL·min^–1^·1.73 m^–2^). Finally, 16 qualified articles (including 1,660 patients) (Mathur et al., 2006; de Brito-Ashurst et al., 2009; Mahajan et al., 2010; Bellasi et al., 2016; Yan et al., 2017; Kendrick et al., 2018; Goraya et al., 2019; Alva et al., 2020; Dubey et al., 2020; Melamed et al., 2020; Raphael et al., 2020a; Raphael et al., 2020b; Bohling et al., 2021; Bovée et al., 2021; Kendrick et al., 2023; Mohebbi et al., 2023) were examined to assess the efficacy and safety of sodium bicarbonate in patients with CKD, including kidney-transplant recipients (two studies, 280 patients) and non-transplant patients (14 studies, 1,380 patients). Figure 1 shows the process of literature selection and the reasons for excluding studies, and Table 1 displays the main characteristics of the included studies.

**TABLE 1:** Characteristics of the included studies in the meta-analysis.

Study ID	Single/Multicenter	N	Dose of NAHCO_3_	Control group	Age (years) mean ± SD	Male (%)	Study duration (mon)	Jadad score	Baseline kidney function	Baseline HCO_3_ (mmol/L)	Patients type
Alva 2020	Single	67	600 mg tid; target HCO_3_ levels>23 mmol/L	Standard treatment	18–40:7.5% 41–60:46.3% 61–80:46.3%	71.6	9	2	eGFR:21.8 ± 4.2 mL/min/1.73 m^2^	16.7 ± 6.2	Non-transplant patients with CKD
Bellasi 2016	Multicenter	145	0.5 mmol/kg/d,bid; target HCO_3_ levels 24–28 mmol/L	Standard treatment	65.5 ± 11.4	57	12	3	Ccl:33 ± 14 mL/min	21.4 ± 1.9	Non-transplant patients with CKD
Bohling 2021	Single	40	0.5mEq/kg of lean body weight/d,bid	Placebo	52 ± 17	80.0	4	5	eGFR:75 ± 22 mL/min/1.73 m^2^	23.4 ± 2.0	Kidney Transplant Recipients
Bovée 2021	Multicenter	30	3,000 mg/d	Standard treatment	62 ± 15	78	1	5	eGFR:20.5 ± 5.0 mL/min/1.73 m^2^	21.7 ± 3.3	Non-transplant patients with CKD
de Brito-Ashurst 2009	Single	134	600 mg tid; target HCO_3_ levels>23 mmol/L	Standard treatment	54.8 ± 20	51.5	24	3	Ccl:20.4 ± 49.2 mL/min/1.73m^2^	19.9 ± 15.4	Non-transplant patients with CKD
Dubey 2020	Single	188	0.5mEq/kg/d; target HCO_3_ levels 24–26 mEq/L	Standard treatment	50.2 ± 11.5	71.3	6	5	eGFR:30.4 ± 10.8 mL/min/1.73 m^2^	18.1 ± 2.3	Non-transplant patients with CKD
Goraya 2019	Single	72	0.3mEq/kg/d	Standard treatment	53.8 ± 5.0	44.4	60	1	eGFR:39.6 ± 6.7 mL/min/1.73 m^2^	23 ± 0.6	Non-transplant patients with CKD
Kendrick 2018	Single	40	0.4mEq/kg/d; bid-tid; target HCO_3_ levels≥23 mEq/L	Standard treatment	59 ± 12	50	1.5	4	eGFR:26.0 ± 8.0 mL/min/1.73 m^2^	19.5 ± 2.3	Non-transplant patients with CKD
Kendrick 2023	Single	109	0.5 mEq/kg of lean body weight/d; bid	Placebo	61.7 ± 11.6	49.5	12	5	eGFR:34.9 ± 9.8 mL/min/1.73 m^2^	23.4 ± 2.2	Non-transplant patients with CKD
Mahajan 2010	Single	80	0.5 mEq/kg of lean body weight/d	Placebo	51.3 ± 8.3	48	60	4	eGFR:75.5 ± 6.1 mL/min/1.73 m^2^	26.1 ± 0.8	Non-transplant patients with CKD
Mathur 2006	Single	40	1.2mEq/kg/d; tid; target HCO_3_ levels 22–26 mEq/L	Placebo	40.5 ± 14.3	62.5	3	3	Serum creatinine: 2.9 ± 1.0 mg/dL	19.4 ± 4.6	Non-transplant patients with CKD
Melamed 2020	Multicenter	149	0.4mEq/kg/d	Placebo	61.0 ± 12.6	46.3	24	5	eGFR:36.3 ± 11.2 mL/min/1.73 m^2^	24.0 ± 2.2	Non-transplant patients with CKD
Mohebbi 2023	Multicenter	242	1.5–4.5 g/d	Placebo	55.5 ± 13.5	69.6	24	5	eGFR:47.9 ± 16.0 mL/min/1.73 m^2^	21.1 ± 2.7	Kidney Transplant Recipients
Raphael 2020A	Single	74	0.5 mEq/kg of lean body weight/d; bid	Placebo	72 ± 8	97	6	5	eGFR:51 ± 18 mL/min/1.73 m^2^	24 ± 2	Non-transplant patients with CKD
Raphael 2020B	Multicenter	194	higher-dose:0.8 meq/kg of lean body weight/d; lower-dose: 0.5 meq/kg of lean body weight/d	Placebo	67 ± 12	67	7	5	eGFR:36 ± 9 mL/min/1.73 m^2^	24 ± 2	Non-transplant patients with CKD
Yan 2017	Single	84	1–2 g/d; target HCO_3_ levels 22–27mEq/L	Placebo	53.1 ± 7.5	58.3	4.5	3	eGFR:18.8 ± 2.1 mL/min/1.73 m^2^	16.3 ± 1.3	Non-transplant patients with CKD

bid:bis in die; tid:ter in die.

### 3.2 Assessment of the risk of bias of the included trials

**FIGURE 2:**
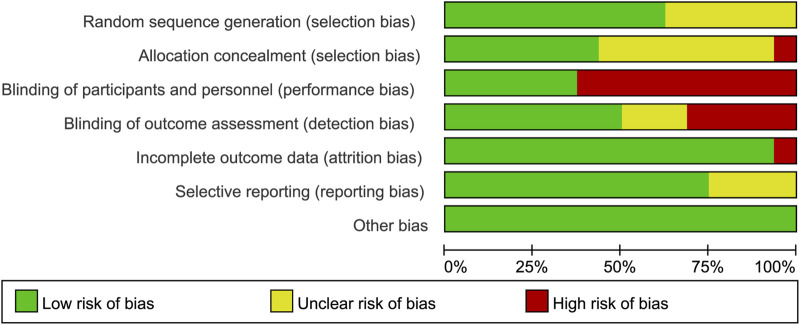
Risk-of-bias summary of the included randomized trials determine using the Cochrane Risk-of-Bias tool.

The Cochrane Collaboration tool was used to assess the risk of bias of the included trials. As shown in Figure 2, only one of the included RCTs was considered to have an overall low risk of bias. Some studies had unclear risks in terms of random sequence generation and allocation concealment because the specific methods used were not reported. Ten trials had a high risk in terms of blinding participants and personnel because they did not use double-blinded methods. In terms of blinding of the outcome assessment, some articles did not provide detailed information, and the outcome evaluators were not blinded in some studies, leading to an unclear or high-risk bias. One study had a high risk for incomplete outcome data, and four studies had an unclear risk for selective reporting. In terms of other biases, all trials were assessed as having a low risk.

### 3.3 Meta-analysis

#### 3.3.1 Changes in kidney function (eGFR or Ccl) from baseline to the end of the study

**FIGURE 3:**
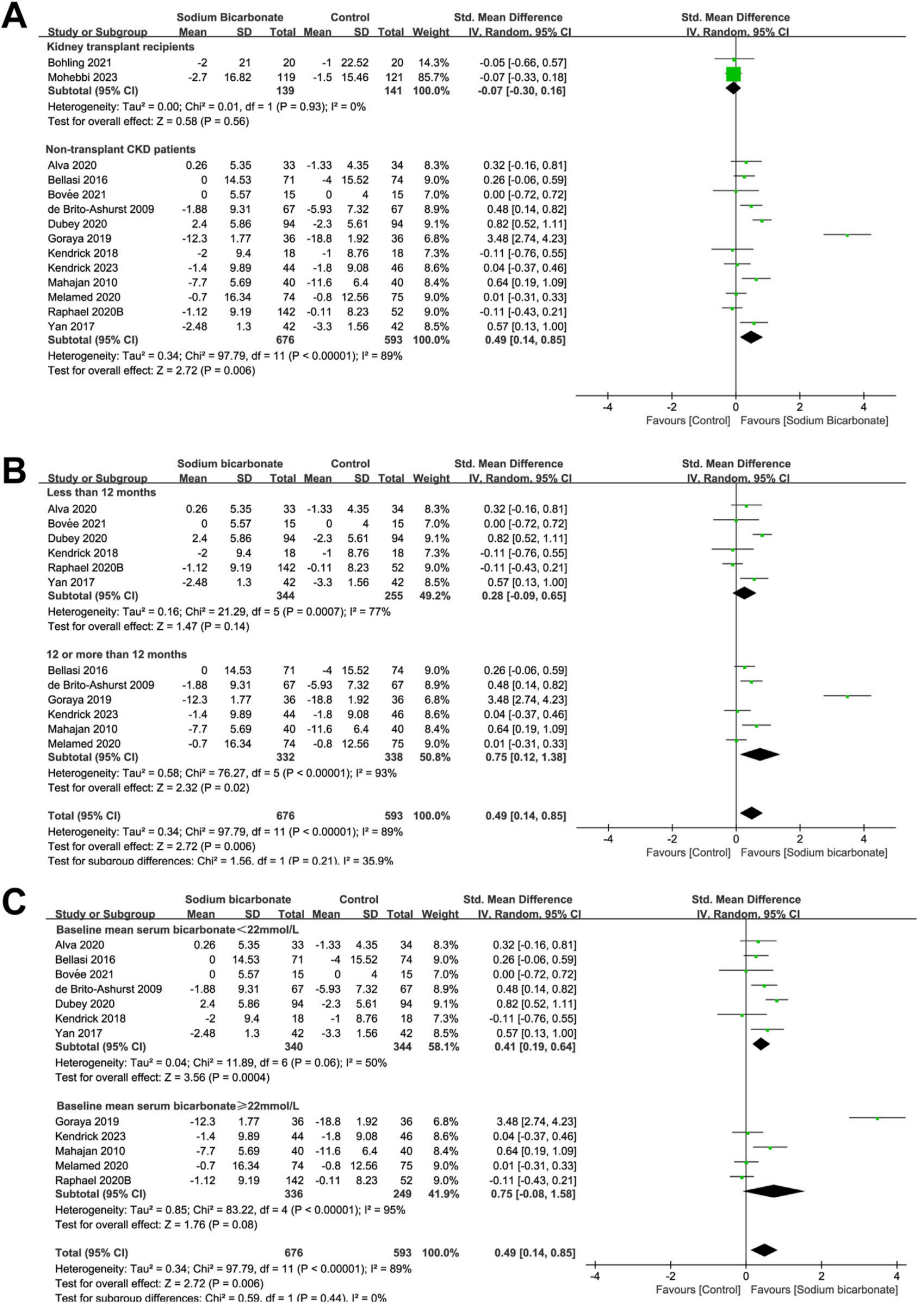
Meta-analysis results of sodium bicarbonate in terms of kidney function (eGFR or Ccl). **(A)** Kidney function (eGFR or Ccl). **(B)** Subgroup analysis of the effect of sodium bicarbonate on kidney function as a function of the study duration. **(C)** Subgroup analysis of the effect of sodium bicarbonate on kidney function according to the baseline mean serum-bicarbonate levels.

As shown in Figure 3A, the effects of sodium bicarbonate on kidney function (eGFR or Ccl) were compared with control treatment (placebo or standard treatment) in two studies of kidney-transplant recipients (n = 280). No significant difference was observed in the kidney function (eGFR or Ccl) between the sodium bicarbonate and control groups (SMD: -0.07 [95% CI: -0.30–0.16], *p* = 0.56; heterogeneity: I^2^ = 0%, *p* = 0.93). In 12 studies involving 1,269 non-transplant patients with CKD, the effects of sodium bicarbonate on kidney function were compared with control groups. The pooled results revealed significant higher kidney function (eGFR or Ccl) levels in the sodium bicarbonate group than in the control group (SMD: 0.49 [95% CI 0.14–0.85], *p* = 0.006; heterogeneity: I^2^ = 89%, *p* < 0.00001). Owing to the high heterogeneity, we used meta-regression to analyze the source of heterogeneity based on the study duration, Jadad score, type of control group, year of publication, male ratio, severity of kidney function at baseline and baseline mean serum-bicarbonate level (Supplementary Table S3). Univariate (*p* = 0.020) and multivariate (*p* = 0.070) meta-regression analyses showed that the study duration might have led to the high heterogeneity. Subgroup analysis was carried out to assess the effect of the study duration (<12 months or ≥12 months). As shown in Figure 3B, the kidney function (eGFR or Ccl) of patients in studies lasting ≥12 months was significantly higher in the sodium bicarbonate group than in the control group (SMD: 0.75 [95% CI 0.12–1.38], *p* = 0.02; heterogeneity: I^2^ = 93%, *p* < 0.00001), whereas no significant difference was observed between the two groups in studies lasting <12 months (SMD: 0.28 [95% CI -0.09–0.65], *p* = 0.14; heterogeneity: I^2^ = 77%, *p* = 0.0007). To investigate whether the severity of metabolic acidosis affected the efficacy of sodium bicarbonate on kidney function (eGFR or Ccl) levels in non-transplant patients with CKD, subgroup analysis was performed according to the baseline mean serum-bicarbonate level (<22 or ≥22 mmol/L). As shown in Figure 3C, in patients with a baseline mean serum-bicarbonate level of <22 mmol/L, the pooled results revealed significant higher kidney function (eGFR or Ccl) levels in the sodium bicarbonate group than in the control group (SMD: 0.41 [95% CI 0.19–0.64], *p* = 0.0004; heterogeneity: I^2^ = 50%, *p* = 0.06). However, no significant difference was observed in patients with a baseline mean serum-bicarbonate level of ≥22 mmol/L between the groups (SMD: 0.75 [95% CI -0.08–1.58], *p* = 0.08; heterogeneity: I^2^ = 95%, *p* < 0.00001).

#### 3.3.2 Changes in serum-bicarbonate levels from baseline to the end of the study

**FIGURE 4:**
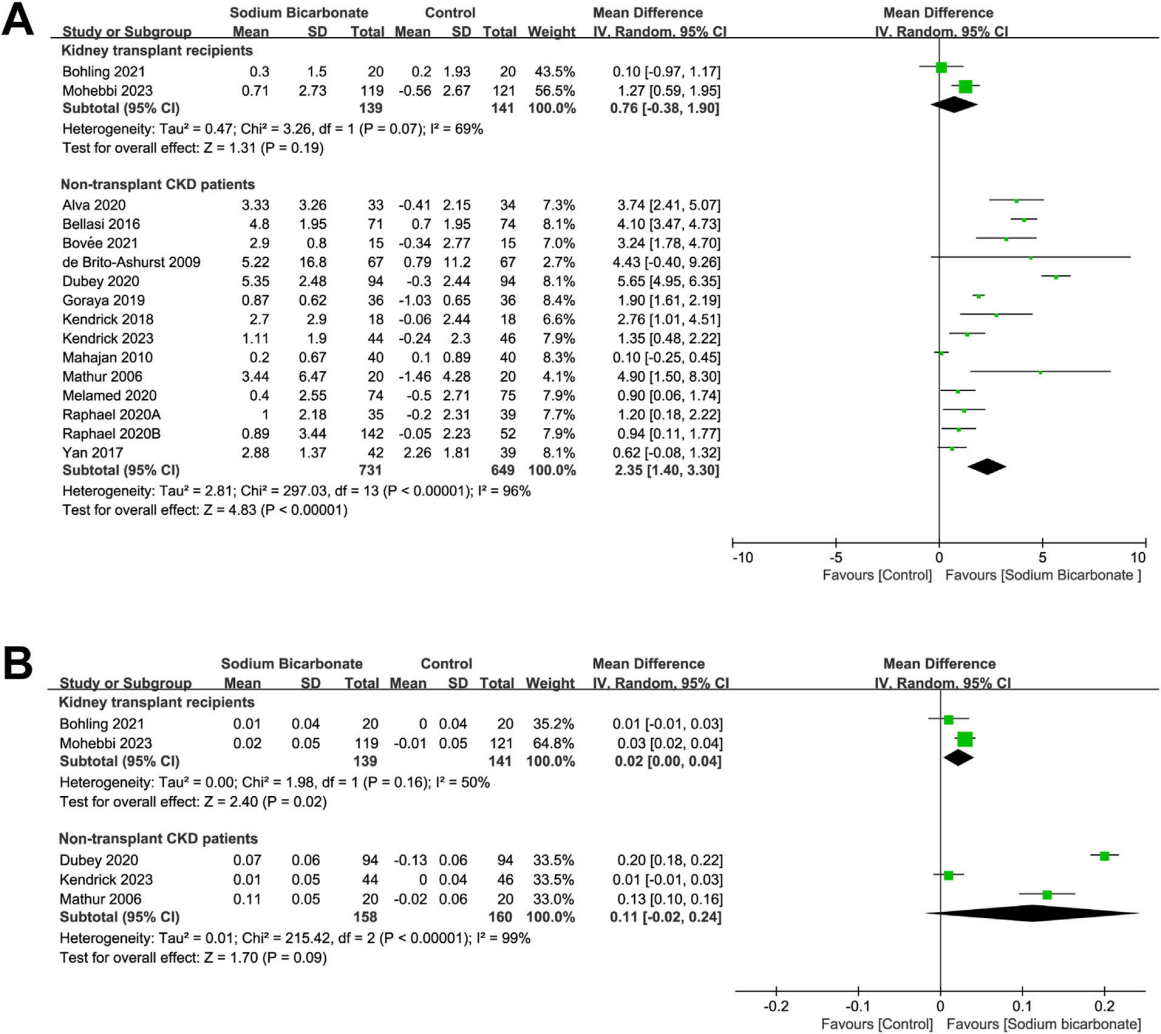
Meta-analysis results of sodium bicarbonate in terms of metabolic acidosis indices. **(A)** Serum bicarbonate. **(B)** Blood pH.

We compared the serum-bicarbonate levels between sodium bicarbonate- and control-treated (placebo or standard treatment) groups found in two studies of kidney-transplant recipients (n = 280). As shown in Figure 4A, no significant difference was observed in the serum-bicarbonate levels between the two groups (MD: 0.76 [95% CI -0.38–1.90), *p* = 0.19; heterogeneity: I^2^ = 69%, *p* = 0.07). We also compared the serum bicarbonate levels observed in 14 studies of sodium bicarbonate- and control-treated groups of patients with CKD who did not undergo a kidney transplant (n = 1,380). The pooled results revealed significant higher serum-bicarbonate levels in the sodium bicarbonate group than in the control group (MD: 2.35 [95% CI 1.40–3.30], *p* < 0.00001; heterogeneity: I^2^ = 96%, *p* < 0.00001). Because we observed high heterogeneity, we carried out meta-regression analyses to analyze the source of the heterogeneity based on the study duration, Jadad score, type of control group, year of publication, male ratio, severity of kidney function at baseline and baseline mean serum-bicarbonate level (Supplementary Table S4). Univariate (*p* = 0.005) and multivariate (*p* = 0.005) meta-regression analyses demonstrated that the type of control group might be a source of heterogeneity. Subgroup analysis was performed based on the type of control group (placebo or standard treatment), as shown in Supplementary Figure S1. The serum-bicarbonate levels of the sodium bicarbonate group were significantly higher than those of the control group in the different subgroups (MD: 3.63 [95% CI 2.18–5.08], *p* < 0.00001; heterogeneity: I^2^ = 95%, *p* < 0.00001 and MD: 0.88 [95% CI 0.34–1.43], *p* = 0.002; heterogeneity: I^2^ = 69%, *p* = 0.004, respectively).

#### 3.3.3 Changes in blood pH from baseline to the end of the study

We compared the effects of sodium bicarbonate treatment with a control (placebo or standard treatment) in terms of the blood pH observed in two studies of kidney-transplant recipients (n = 280). As shown in Figure 4B, the blood pH in the sodium bicarbonate group was significantly higher than that in the control group (MD: 0.02 [95% CI 0.00–0.04], *p* = 0.02; heterogeneity: I^2^ = 50%, *p* = 0.16). In three studies, the effects of sodium bicarbonate and control treatment on blood-pH levels were compared in patients with CKD who did not undergo a kidney transplant (n = 318). No significant difference was observed in the blood-pH levels between the sodium bicarbonate- and control-treated groups (MD: 0.11 [95% CI -0.02–0.24], *p* = 0.09; heterogeneity: I^2^ = 99%, *p* < 0.00001).

#### 3.3.4 Change in systolic blood pressure from baseline to the end of the study

**FIGURE 5:**
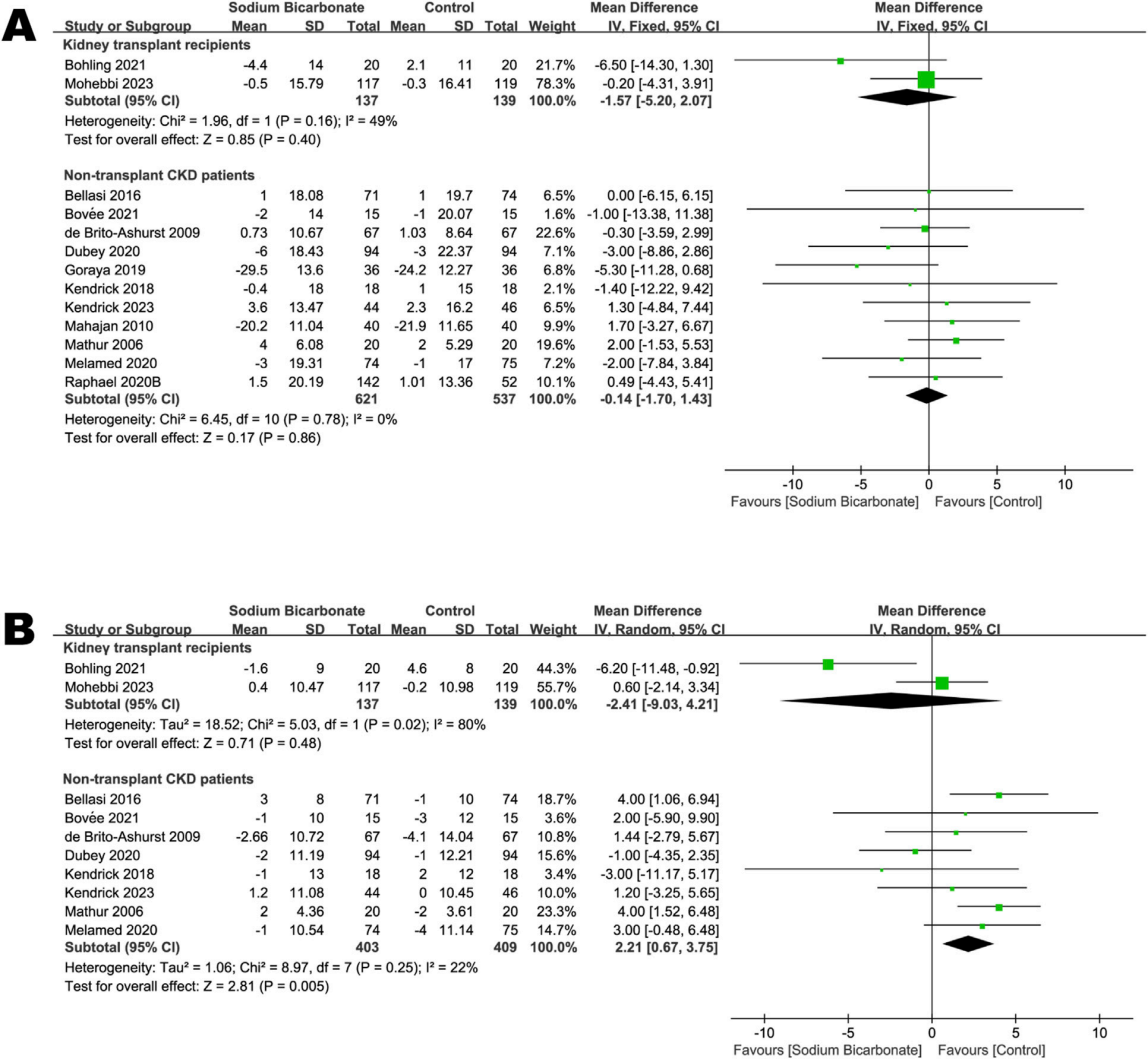
Meta-analysis results of sodium bicarbonate in terms of blood-pressure levels. **(A)** Systolic blood pressure. **(B)** Diastolic blood pressure.

We compared the effects of sodium bicarbonate treatment with a control (placebo or standard therapy) on systolic-blood pressure levels found in two studies of kidney-transplant recipients (n = 276) and in 11 studies of non-transplant patients with CKD (n = 1,158). As shown in Figure 5A, no significant difference was observed in systolic blood pressures between the sodium bicarbonate and control groups in both kidney-transplant recipients and non-transplant patients (MD: -1.57 [95% CI -5.20–2.07], *p* = 0.40; heterogeneity: I^2^ = 49%, *p* = 0.16 and MD: -0.14 [95% CI -1.70–1.43], *p* = 0.86; heterogeneity: I^2^ = 0%, *p* = 0.78, respectively).

#### 3.3.5 Change in diastolic blood pressure from baseline to the end of the study

We compared the effects of sodium bicarbonate treatment with a control (placebo or standard therapy) on diastolic-blood pressure levels found in two studies of kidney-transplant recipients (n = 276). As shown in Figure 5B, no significant difference was observed in the diastolic-blood pressure levels between the two groups (MD: -2.41 [95% CI -9.03–4.21], *p* = 0.48; heterogeneity: I^2^ = 80%, *p* = 0.02). We compared sodium bicarbonate with controls in terms of diastolic blood pressure levels in eight studies of non-transplant patients with CKD (n = 812). The pooled results revealed significantly higher diastolic blood pressures in the sodium bicarbonate group than in the control group (MD: 2.21 [95% CI 0.67–3.75], *p* = 0.005; heterogeneity: I^2^ = 22%, *p* = 0.25). To investigate whether kidney function affected the increased diastolic blood pressure induced by sodium bicarbonate in non-transplant patients with CKD, we conducted subgroup analysis according to the baseline mean eGFR or Ccl, i.e., ≥30 or <30 (mL·min^–1^·1.73 m^–2^) or (mL·min^–1^). As shown in Supplementary Figure S2, the diastolic-blood pressure levels in the sodium bicarbonate group were significantly higher than those in the control group in patients with a baseline mean eGFR or Ccl ≥30 or <30 (mL·min^–1^·1.73 m^–2^) or (mL·min^–1^), as indicated by the following parameters: MD: 3.10 (95% CI 1.09–5.11), *p* = 0.002; heterogeneity: I^2^ = 0%, *p* = 0.59 and MD: 1.86 (95% CI 0.14–3.57), *p* = 0.03; heterogeneity: I^2^ = 43%, *p* = 0.13, respectively.

#### 3.3.6 AEs

**FIGURE 6:**
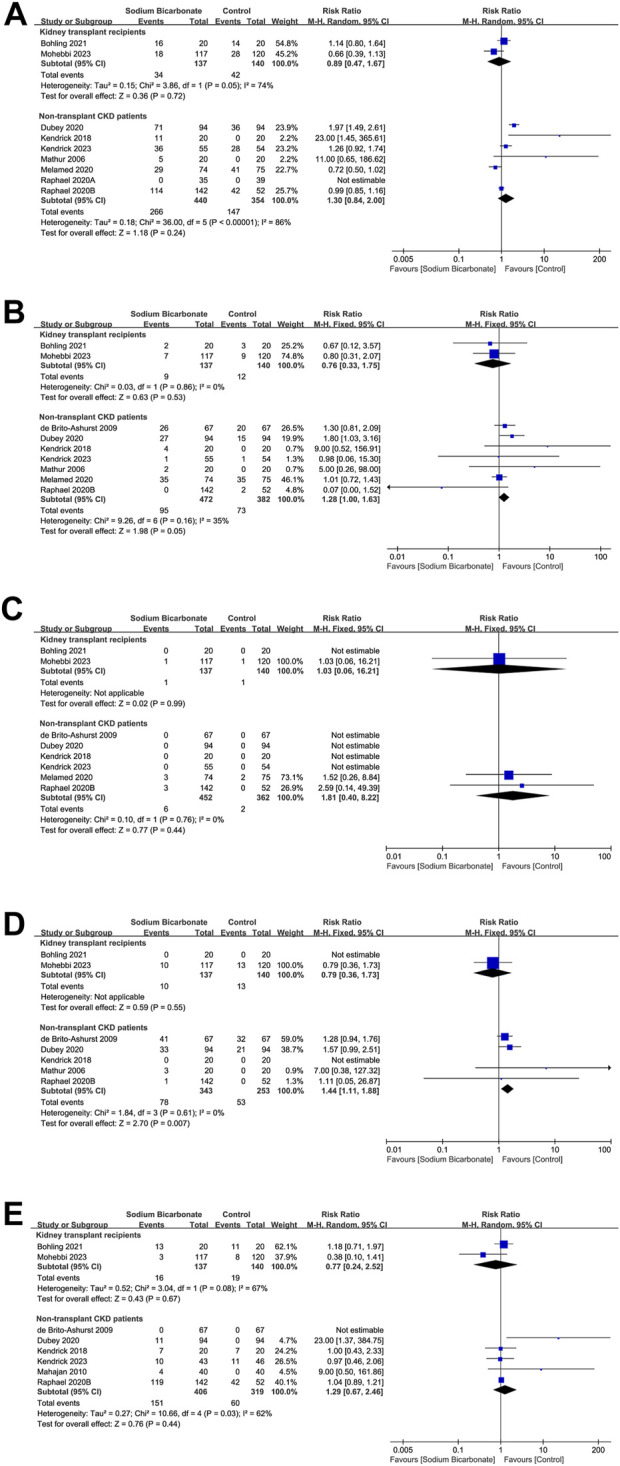
Meta-analysis results of sodium bicarbonate in terms of safety outcomes. **(A)** AEs. **(B)** Edema. **(C)** Heart failure. **(D)** Worsening hypertension. **(E)** Gastrointestinal disorders.

We compared the incidences of AEs between the sodium bicarbonate and control groups (placebo or standard treatment) reported for two studies of kidney-transplant recipients (n = 277) and for seven studies of non-transplant patients with CKD (n = 794). Regarding the non-transplant patients with CKD, no AEs occurred in the two groups reported by (Raphael et al., 2020a). Hence, we included the remaining six studies in our meta-analysis. As shown in Figure 6A, we found no significant difference in AEs between the sodium bicarbonate and control groups with either kidney-transplant recipients or non-transplant patients with CKD (RR: 0.89 [95% CI 0.47–1.67], *p* = 0.72; heterogeneity: I^2^ = 74%, *p* = 0.05 and RR: 1.30 [95% CI 0.84–2.00], *p* = 0.24; heterogeneity: I^2^ = 86%, *p* < 0.00001, respectively).

#### 3.3.7 Edema

We compared the effects of sodium bicarbonate treatment with a control (placebo or standard therapy) on the risk of edema reported for two studies of kidney-transplant recipients (n = 277). As shown in Figure 6B, no significant difference was found in the risk for edema between the sodium bicarbonate and control groups (RR: 0.76 [95% CI 0.33–1.75], *p* = 0.53; heterogeneity: I^2^ = 0%, *p* = 0.86). We also compared the risk of edema between the sodium bicarbonate and control groups reported for seven studies of non-transplant patients with CKD (n = 854). The incidence of edema was higher in the sodium bicarbonate group than in the control group (RR: 1.28 [95% CI 1.00–1.63], *p* = 0.05; heterogeneity: I^2^ = 35%, *p* = 0.16). To investigate whether kidney function affected the incidence of edema caused by sodium bicarbonate in non-transplant patients with CKD, we conducted subgroup analysis according to the baseline mean eGFR or Ccl (≥30 or <30 [mL·min^–1^·1.73 m^–2^] or [mL·min^–1^]). As shown in Supplementary Figure S3, the incidence of edema in the sodium bicarbonate group was significantly higher than that in the control group for patients with a baseline mean eGFR or Ccl of <30 (mL·min^–1^·1.73 m^–2^) or (mL·min^–1^), based on the following parameters: RR: 1.67 (95% CI 1.17–2.38), *p* = 0.005; heterogeneity: I^2^ = 0%, and *p* = 0.39. However, no significant difference was observed in patients with a baseline mean eGFR or Ccl of ≥30 (mL·min^–1^·1.73 m^–2^) or (mL·min^–1^), according to these parameters: RR: 0.93 (95% CI 0.66–1.29), *p* = 0.65; heterogeneity: I^2^ = 32%, and *p* = 0.23.

#### 3.3.8 Heart failure

We compared data reported in terms of the effect of sodium bicarbonate on the incidence of heart failure compared to controls (placebo or standard treatment) in two studies of kidney-transplant recipients (n = 277). As shown in Figure 6C, no heart failure events occurred in the two groups reported by (Bohling et al., 2021); thus, we excluded those data from our meta-analysis. The results revealed no difference in the incidence of heart failure between the sodium bicarbonate and control groups (RR: 1.03 [95% CI 0.06–16.21], *p* = 0.99). By analyzing the results of six studies that compared the risk of heart failure between sodium bicarbonate and controls in non-transplant patients with CKD (n = 814), we found that no such events occurred in the groups (as reported in four studies). Hence, the remaining two studies were ultimately included in our meta-analysis, and the pooled results revealed no difference in the incidence of heart failure between the sodium bicarbonate and control groups (RR: 1.81 [95% CI 0.40–8.22], *p* = 0.44; heterogeneity: I^2^ = 0%, *p* = 0.76).

#### 3.3.9 Worsening hypertension

We compared data regarding the effect of sodium bicarbonate treatment or controls (placebo or standard therapy) on the incidence of worsening hypertension reported for two studies of kidney-transplant recipients (n = 277). As shown in Figure 6D, no events occurred in the two groups reported by (Bohling et al., 2021), and those data were excluded from our meta-analysis. The results revealed that no difference occurred in the incidence of worsening hypertension between sodium bicarbonate- and control-treated groups (RR: 0.79 [95% CI 0.36–1.73], *p* = 0.55). We also compared the incidence of worsening hypertension between the sodium bicarbonate and control groups reported for five studies of non-transplant patients with CKD (n = 596); no such events were reported by (Kendrick et al., 2018). Hence, the remaining four studies were ultimately included in the meta-analysis. The pooled results demonstrated that the incidence of worsening hypertension was higher in the sodium bicarbonate group than in the control group (RR: 1.44 [95% CI 1.11–1.88], *p* = 0.007; heterogeneity: I^2^ = 0%, *p* = 0.61).

#### 3.3.10 Gastrointestinal disorders

We compared the incidence of gastrointestinal disorders between the sodium bicarbonate and control groups (placebo or standard treatment) reported for two studies of kidney-transplant recipients (n = 277) and six studies of non-transplant patients with CKD (n = 725). With the non-transplant patients with CKD, no such events occurred in the two groups reported by (de Brito-Ashurst et al., 2009). The remaining five studies were ultimately included in our meta-analysis. As shown in Figure 6E, we observed no significant difference in the incidence of gastrointestinal disorders between sodium bicarbonate- and control-treated groups, with either kidney-transplant recipients or non-transplant patients with CKD (RR: 0.77 [95% CI 0.24–2.52], *p* = 0.67; heterogeneity: I^2^ = 67%, *p* = 0.08 and RR: 1.29 [95% CI 0.67–2.46], *p* = 0.44; heterogeneity: I^2^ = 62%, *p* = 0.03, respectively).

#### 3.3.11 Sensitivity analysis

For non-transplant patients with CKD, sensitivity analyses were performed using the one-by-one elimination method to evaluate outcome stabilities. Most outcomes were stable, except for the blood pH and occurrence of edema (Supplementary Tables S5–S13).

#### 3.3.12 Publication bias

Outcomes that included over 10 study comparisons were assessed for publication bias. No significant evidence of publication bias was found using Egger’s test in terms of an asymmetrical funnel plot (Supplementary Figures S4-S9; Supplementary Table S14).

#### 3.3.13 GRADE approach for assessing outcomes

We assessed all outcomes using the GRADE approach (https://www.gradepro.org/) in terms of the risk of bias, inconsistencies, indirectness, imprecision, and other considerations (including publication bias, large effect, plausible confounding, and a dose–response gradient). Our comprehensive analysis indicated that all outcomes were of low or very-low quality (Supplementary Table S15).

## 4 Discussion

Our systematic review and meta-analysis demonstrated that oral sodium bicarbonate might delay the decline of kidney function in non-transplant patients studied for ≥12 months or with a baseline mean bicarbonate level of <22 mmol/L, but that it did not preserve graft function in kidney-transplant recipients. Oral sodium bicarbonate corrected metabolic acidosis in kidney-transplant recipients and non-transplant patients with CKD. In general, for kidney-transplant recipients and non-transplant patients with CKD, the incidence of AEs in the sodium bicarbonate group was comparable to that in the control group; however, some safety concerns were still noted. In non-transplant patients with CKD, oral sodium bicarbonate may increase the diastolic blood pressure and incidences of worsening hypertension and edema, whereas no significant effects were observed on systolic blood pressure, heart failure, or gastrointestinal disorders. No significant safety concerns were observed among kidney-transplant recipients. All the results of our study were rated as providing low-quality or very low-quality evidence.

For non-transplant patients with CKD, oral sodium bicarbonate may slow the decline in kidney function, consistent with the results of previous meta-analyses (Navaneethan et al., 2019; Cheng et al., 2021; Hultin et al., 2021) but differing with findings in kidney-transplant recipients in our meta-analysis. A natural question is why sodium bicarbonate has different effects on kidney function in kidney-transplant recipients and non-transplant patients with CKD.

First of all, the pathophysiological mechanisms underlying metabolic acidosis in kidney-transplant recipients appear to differ from those in non-transplant patients with CKD. The kidneys serve essential roles in maintaining the acid–base balance by reabsorbing bicarbonate, regenerating bicarbonate through ammoniagenesis, and excreting acid (Wagner et al., 2019). The degree of nephron loss with reduced ammoniagenesis (the main cause of metabolic acidosis in non-transplant patients with CKD) was similar to that observed in kidney-transplant recipients. However, other factors particular to kidney-transplant recipients may promote and change the phenotype of metabolic acidosis, which is typically manifested as renal tubular acidosis (Ritter and Mohebbi, 2020). These factors include tubular damage, hyperparathyroidism (Bank and Aynediian, 1976; Better, 1980; Yakupoglu et al., 2007), calcineurin inhibitors (such as tacrolimus (Heering et al., 1998; Schwarz et al., 2006; Mohebbi et al., 2009; George et al., 2022) and cyclosporine A (Heering and Grabensee, 1991; Watanabe et al., 2005; Blankenstein et al., 2017)), immunological injury from graft rejection (Mookerjee et al., 1969; Batlle et al., 1981; Cho et al., 2003), the donation process, donor-related characteristics (such as deceased donor transplantation) (Keven et al., 2007; Tariq and Dobre, 2022), recipient-related factors (Tariq and Dobre, 2022), insulin resistance (Ambühl, 2007; Messa et al., 2016), high dietary acid intake (van den Berg et al., 2012), higher urine flow with the single (transplanted) kidney (Chan et al., 1974; Ambühl, 2007), other medications used after transplantation (such as the antibiotic trimethoprim-sulfamethoxazole) (Pochineni and Rondon-Berrios, 2018), and hyperkalemia after kidney transplantation (Harris et al., 2018). Sodium bicarbonate treatment might not be able to overcome the pathophysiological mechanisms caused by the above-mentioned specific factors (especially calcineurin inhibitors) to improve acid retention in the blood, interstitium, and cells of the transplanted kidney (Kuhn et al., 2024).

Second, the study duration, serum-bicarbonate levels at the end of the study, masking, and kidney-function measures (which differed widely in studies on kidney-transplant recipients and non-transplant patients with CKD) may have contributed to the inconsistent results. Among these factors, the study duration and serum-bicarbonate levels at the end of the study impacted the results most. Subgroup analysis of kidney function based on the study duration in our meta-analysis showed that, in non-transplant patients with CKD, sodium bicarbonate delayed the decline of kidney function in patients studied for ≥12 months, whereas it might not have played a positive role for patients in studies lasting <12 months. Owing to the more complex pathophysiological mechanisms of kidney-transplant recipients, one of the included studies was relatively short (Mirioglu and Frangou, 2023), and kidney-transplant recipients may need a longer treatment time for graft-function preservation. The results of a retrospective study demonstrated that elevated serum-bicarbonate levels correlated favorably with long-term transplant and patient survival in kidney-transplant recipients (Wiegand et al., 2022). The results of another recent study of a transplant cohort showed that even an increase of a 1 mmol/L in serum-bicarbonate level significantly improved graft and patient survival and that, even in patients with normal serum-bicarbonate levels, an elevated bicarbonate level was linked to a better prognosis (Mathur et al., 2023). The lowest risks of graft failure and mortality were associated with bicarbonate concentrations between 26 and 28 mmol/L (Park et al., 2017). These findings suggest that kidney-transplant recipients require higher bicarbonate concentrations for graft-function preservation. In contrast, the mean bicarbonate concentrations at the endpoints of two included studies on kidney-transplant recipients were approximately 24 and 22 mmol/L, respectively. However, higher sodium bicarbonate concentrations require higher doses of oral sodium bicarbonate and the resulting burdens in terms of the medication and side effects should be considered.

Third, in two included studies on kidney-transplant recipients, the effects of sodium bicarbonate on faster graft function decline were not analyzed. Whether sodium bicarbonate plays a positive role in preserving kidney function in such patients needs to be investigated. Fourth, the results of a previous study demonstrated that alkali therapy may protect renal function by restraining the local proliferation of immune cells, a mechanism that is independent of improving traditional renal fibrosis and atrophy (Pastor Arroyo et al., 2022), whereas the application of immunosuppressants in kidney-transplant recipients may have impaired the process.

Finally, we performed subgroup analysis of kidney function in non-transplant patients with CKD based on the baseline mean serum-bicarbonate levels (<22 or ≥22 mmol/L). In our meta-analysis, we found that sodium bicarbonate could delay kidney-function decline in patients with a baseline mean serum bicarbonate of <22 mmol/L, whereas it might not have played a positive role in patients with a baseline mean serum bicarbonate of ≥22 mmol/L. Therefore, metabolic acidosis in kidney-transplant recipients was relatively mild (Mirioglu and Frangou, 2023) in both included studies, and the baseline mean serum bicarbonate in one of the included studies was normal (≥22 mmol/L). Whether sodium bicarbonate affects renal function in kidney-transplant recipients with severe metabolic acidosis remains unclear; thus, the included population should be expanded for further verification purposes.

In this study, we investigated the safety of sodium bicarbonate. Although no significant safety concerns were observed in kidney-transplant recipients, more emphasis should be placed on the AEs caused by sodium bicarbonate because it may increase diastolic-blood pressure levels and the incidence of worsening hypertension and edema observed in non-transplant patients with CKD. In terms of heart failure and gastrointestinal reactions, no significant differences were observed between the sodium bicarbonate-intervention and control groups, consistent with the results of a previous meta-analysis (Cheng et al., 2021; Hultin et al., 2021; Beynon-Cobb et al., 2023). Sodium bicarbonate may lead to an increased incidence of worsening hypertension and edema, which is generally consistent with one of the previous meta-analyses (Navaneethan et al., 2019) but different from another (Hultin et al., 2021). Interestingly, our meta-analysis revealed a key difference from previous meta-analyses (Navaneethan et al., 2019; Cheng et al., 2021; Hultin et al., 2021; Beynon-Cobb et al., 2023): we found that sodium bicarbonate may increase the diastolic blood pressure rather than the systolic blood pressure, which is in line with the findings of a recently published study performed to investigate the impact of the sodium bicarbonate load on blood pressure in an experimental model of CKD (Mannon et al., 2024). The increased incidence of edema, worsening of hypertension, and higher diastolic pressure induced by sodium bicarbonate may be related to sodium-mediated fluid retention.

Moreover, we analyzed the reasons for inconsistent results regarding the safety of sodium bicarbonate in kidney-transplant recipients and non-transplant patients with CKD. In the non-transplant patients, our subgroup meta-analysis of diastolic blood pressures based on the baseline mean eGFR or Ccl revealed significant increases in diastolic blood pressure, both in patients with a baseline mean eGFR or Ccl of ≥30 or <30 (mL·min^–1^·1.73 m^–2^) or (mL·min^–1^). We could not perform subgroup analyses based on a baseline mean eGFR or Ccl of ≥45 due to limitations in the included studies. However, in terms of the incidence of edema, patients with baseline mean eGFR or Ccl <30 (mL·min^–1^·1.73 m^–2^) or (mL·min^–1^) showed a significant higher incidence of edema after treatment with sodium bicarbonate, whereas patients with a baseline mean eGFR or Ccl of ≥30 did not, which might explain the unstable outcome in terms of the incidence of edema. These results suggest an important insight that kidney function may explain the inconsistent results regarding the safety of sodium bicarbonate in kidney-transplant recipients and non-transplant patients with CKD found in our study. The baseline mean eGFR or Ccl of kidney-transplant recipients was >45 in two included studies, and the mean eGFR or Ccl was >60 in one study. Of the 14 studies that included non-transplant patients with CKD, only one study (Mahajan et al., 2010) involved a baseline mean eGFR or Ccl of ≥45. Previous findings have shown that, as kidney function decreases, excretory function gradually worsens and promotes greater salt sensitivity (Brenner and Anderson, 1992), resulting in volume retention and elevated blood pressure (Mannon et al., 2024). Therefore, we believe that sodium bicarbonate may cause the above-mentioned AEs in both kidney-transplant recipients and non-transplant patients with CKD. The most worrisome of these AEs is increased blood pressure because hypertension is considered a cardiovascular risk factor in CKD (Burnier and Damianaki, 2023), and even minor changes in blood pressure might raise the risk of adverse cardiovascular effects and CKD progression (Chang et al., 2019). Therefore, the AEs of sodium bicarbonate merit greater consideration.

The findings of one study demonstrated that sodium bicarbonate supplementation did not elevate blood pressure or cause Na^+^ retention when NaCl intake was severely restricted in patients with CKD (Bushinsky, 2019). When the total Na^+^ intake was maintained within the capacity of the remaining kidney to excrete Na^+^, the blood pressure and volume retention did not increase (Mannon et al., 2024). Consequently, dietary interventions including strict NaCl restriction or even free NaCl, base-producing vegetables and fruits, lower animal protein intake (Goraya et al., 2019; Sotomayor et al., 2020), and other emerging drugs such as veverimer (Wesson et al., 2019), should benefit kidney-transplant recipients and non-transplant patients with CKD.

However, recent data showed that sodium bicarbonate was linked to a lower risk of serious adverse cardiovascular events, such as heart failure, myocardial infarction, and stroke, in patients with advanced CKD (Cheng et al., 2023), although those findings were inconsistent with our meta-analysis of the CKD stage in our study. Besides, sodium bicarbonate delayed the progression of kidney function (Yan et al., 2017; Alva et al., 2020), increased serum klotho levels (Raphael et al., 2024), improved nutritional status (Szczecińska et al., 2022), muscle mass (Dubey et al., 2020), insulin resistance (Bellasi et al., 2016) and vascular endothelial function (Kendrick et al., 2018) in non-transplant patients with CKD. The results of another study showed that kidney-transplant recipients with metabolic acidosis exhibited kidney transcriptome abnormalities that were partially recovered by sodium bicarbonate supplementation (Imenez Silva et al., 2021). In addition, some researchers have proposed that alkali therapy is a well-tolerated, safe, and cost-effective treatment that can slow the progression of graft failure, thereby extending long-term graft survival (Banhara et al., 2015). These results provide some evidence for the potential benefits of sodium bicarbonate in kidney-transplant recipients and non-transplant patients with CKD; however, further large RCT trials are needed to verify this finding.

This study was the first to simultaneously evaluate the efficacy and safety of oral sodium bicarbonate in kidney-transplant recipients and non-transplant patients with stage 1–4 CKD. However, this study has some limitations. First, high heterogeneity was noted among the studies. Although we analyzed the source of heterogeneity in terms of three limited variables, we could not assess other clinical heterogeneities, such as the participant characteristics, owing to the lack of individual participant data. Second, the study duration, masking, type of control treatments, sodium bicarbonate dosage, and kidney-function measures were designed differently between the studies. Third, the short study duration in many of the included studies was not convincing enough to confirm the results for chronic diseases. Fourth, changes in kidney function were not a primary or secondary outcome for the included trials, which undermined the confidence in the results. Finally, the number of large-scale studies examined in this study was not sufficient, especially for kidney-transplant recipients, with only two RCTs included. Therefore, the results of this study need to be verified in several large-scale international studies.

In conclusion, we found that oral sodium bicarbonate may slow kidney-function decline in non-transplant patients with CKD undergoing sodium bicarbonate supplementation for ≥12 months or with a baseline serum bicarbonate level of <22 mmol/L, although it might not preserve graft function in kidney-transplant recipients. Sodium bicarbonate improved metabolic acidosis in both kidney-transplant recipients and non-transplant patients with CKD. However, we observed some safety concerns regarding increased diastolic pressure and increased incidences of worsening hypertension and edema induced by sodium bicarbonate, which should be considered. The potential adverse cardiovascular effects of sodium bicarbonate in patients with CKD should be evaluated before starting treatment, and dietary interventions and blood-pressure monitoring should be performed during treatment. More powerful RCTs are required to investigate the benefits and risks of sodium bicarbonate and determine the optimal sodium bicarbonate treatment strategies for kidney-transplant recipients and non-transplant patients with CKD. Furthermore, mechanistic differences in metabolic acidosis (especially the effects of calcineurin inhibitors) between kidney-transplant recipients and non-transplant patients with CKD, and the renal-protective mechanisms with sodium bicarbonate need to be explored.

## Data Availability

The original contributions presented in the study are included in the article/Supplementary Material, further inquiries can be directed to the corresponding author.

## Abbreviations

AE, adverse effect; Ccl, creatinine clearance; CI, confidence interval; CKD, chronic kidney disease; eGFR, estimated glomerular filtration rate; ESRD, end-stage renal disease; GRADE, Grading of Recommendations, Assessment, Development, and Evaluation; KDIGO, Kidney Disease: Improving Global Outcomes; MD, mean difference; PRISMA, Preferred Reporting Items for Systematic Reviews and Meta-Analyses; RCT, randomized controlled trial; RE, random effect; RR, risk ratio; SMD, standardized mean difference.
