# Supplementary material for: Efficacy and safety of oral sodium bicarbonate in kidney-transplant recipients and non-transplant patients with chronic kidney disease: a systematic review and meta-analysis
Source: Front Pharmacol. 2024 Aug 26;15:1411933. doi: 10.3389/fphar.2024.1411933 (PMC11381255; doi:10.3389/fphar.2024.1411933)

Supplementary Material

Efficacy and Safety of Oral Sodium Bicarbonate in Kidney-Transplant Recipients and Non-Transplant Patients with Chronic Kidney Disease: a Systematic Review and Meta-Analysis

Yun Wu^†^, Ying Wang^†^, Weijun Huang, Xi Guo, Baoluo Hou, Jingyi Tang, Yuqi Wu, Huijuan Zheng, Yanling Pan, Wei Jing Liu*

*** Correspondence:** Wei Jing Liu: [liuweijing-1977@hotmail.com](mailto:liuweijing-1977@hotmail.com,)

# Supplementary Tables

- 1. **Supplementary Table 1 (PRISMA 2020 checklist)**

| **Section and Topic** | **Item #** | **Checklist item** | **Location where item is reported** |
| --- | --- | --- | --- |
| **TITLE** | | |  |
| Title | 1 | Identify the report as a systematic review. |  |
| **ABSTRACT** | | |  |
| Abstract | 2 | See the PRISMA 2020 for Abstracts checklist. |  |
| **INTRODUCTION** | | |  |
| Rationale | 3 | Describe the rationale for the review in the context of existing knowledge. |  |
| Objectives | 4 | Provide an explicit statement of the objective(s) or question(s) the review addresses. |  |
| **METHODS** | | |  |
| Eligibility criteria | 5 | Specify the inclusion and exclusion criteria for the review and how studies were grouped for the syntheses. |  |
| Information sources | 6 | Specify all databases, registers, websites, organisations, reference lists and other sources searched or consulted to identify studies. Specify the date when each source was last searched or consulted. |  |
| Search strategy | 7 | Present the full search strategies for all databases, registers and websites, including any filters and limits used. |  |
| Selection process | 8 | Specify the methods used to decide whether a study met the inclusion criteria of the review, including how many reviewers screened each record and each report retrieved, whether they worked independently, and if applicable, details of automation tools used in the process. |  |
| Data collection process | 9 | Specify the methods used to collect data from reports, including how many reviewers collected data from each report, whether they worked independently, any processes for obtaining or confirming data from study investigators, and if applicable, details of automation tools used in the process. |  |
| Data items | 10a | List and define all outcomes for which data were sought. Specify whether all results that were compatible with each outcome domain in each study were sought (e.g. for all measures, time points, analyses), and if not, the methods used to decide which results to collect. |  |
|  | 10b | List and define all other variables for which data were sought (e.g. participant and intervention characteristics, funding sources). Describe any assumptions made about any missing or unclear information. |  |
| Study risk of bias assessment | 11 | Specify the methods used to assess risk of bias in the included studies, including details of the tool(s) used, how many reviewers assessed each study and whether they worked independently, and if applicable, details of automation tools used in the process. |  |
| Effect measures | 12 | Specify for each outcome the effect measure(s) (e.g. risk ratio, mean difference) used in the synthesis or presentation of results. |  |
| Synthesis methods | 13a | Describe the processes used to decide which studies were eligible for each synthesis (e.g. tabulating the study intervention characteristics and comparing against the planned groups for each synthesis (item #5)). |  |
|  | 13b | Describe any methods required to prepare the data for presentation or synthesis, such as handling of missing summary statistics, or data conversions. |  |
|  | 13c | Describe any methods used to tabulate or visually display results of individual studies and syntheses. |  |
|  | 13d | Describe any methods used to synthesize results and provide a rationale for the choice(s). If meta-analysis was performed, describe the model(s), method(s) to identify the presence and extent of statistical heterogeneity, and software package(s) used. |  |
|  | 13e | Describe any methods used to explore possible causes of heterogeneity among study results (e.g. subgroup analysis, meta-regression). |  |
|  | 13f | Describe any sensitivity analyses conducted to assess robustness of the synthesized results. |  |
| Reporting bias assessment | 14 | Describe any methods used to assess risk of bias due to missing results in a synthesis (arising from reporting biases). |  |
| Certainty assessment | 15 | Describe any methods used to assess certainty (or confidence) in the body of evidence for an outcome. |  |
| **RESULTS** | | |  |
| Study selection | 16a | Describe the results of the search and selection process, from the number of records identified in the search to the number of studies included in the review, ideally using a flow diagram. |  |
|  | 16b | Cite studies that might appear to meet the inclusion criteria, but which were excluded, and explain why they were excluded. |  |
| Study characteristics | 17 | Cite each included study and present its characteristics. |  |
| Risk of bias in studies | 18 | Present assessments of risk of bias for each included study. |  |
| Results of individual studies | 19 | For all outcomes, present, for each study: (a) summary statistics for each group (where appropriate) and (b) an effect estimate and its precision (e.g. confidence/credible interval), ideally using structured tables or plots. |  |
| Results of syntheses | 20a | For each synthesis, briefly summarise the characteristics and risk of bias among contributing studies. |  |
|  | 20b | Present results of all statistical syntheses conducted. If meta-analysis was done, present for each the summary estimate and its precision (e.g. confidence/credible interval) and measures of statistical heterogeneity. If comparing groups, describe the direction of the effect. |  |
|  | 20c | Present results of all investigations of possible causes of heterogeneity among study results. |  |
|  | 20d | Present results of all sensitivity analyses conducted to assess the robustness of the synthesized results. |  |
| Reporting biases | 21 | Present assessments of risk of bias due to missing results (arising from reporting biases) for each synthesis assessed. |  |
| Certainty of evidence | 22 | Present assessments of certainty (or confidence) in the body of evidence for each outcome assessed. |  |
| **DISCUSSION** | | |  |
| Discussion | 23a | Provide a general interpretation of the results in the context of other evidence. |  |
|  | 23b | Discuss any limitations of the evidence included in the review. |  |
|  | 23c | Discuss any limitations of the review processes used. |  |
|  | 23d | Discuss implications of the results for practice, policy, and future research. |  |
| **OTHER INFORMATION** | | |  |
| Registration and protocol | 24a | Provide registration information for the review, including register name and registration number, or state that the review was not registered. |  |
|  | 24b | Indicate where the review protocol can be accessed, or state that a protocol was not prepared. |  |
|  | 24c | Describe and explain any amendments to information provided at registration or in the protocol. |  |
| Support | 25 | Describe sources of financial or non-financial support for the review, and the role of the funders or sponsors in the review. |  |
| Competing interests | 26 | Declare any competing interests of review authors. |  |
| Availability of data, code and other materials | 27 | Report which of the following are publicly available and where they can be found: template data collection forms; data extracted from included studies; data used for all analyses; analytic code; any other materials used in the review. |  |

*From:*  Page MJ, McKenzie JE, Bossuyt PM, Boutron I, Hoffmann TC, Mulrow CD, et al. The PRISMA 2020 statement: an updated guideline for reporting systematic reviews. BMJ 2021;372:n71. doi: 10.1136/bmj.n71

- 1. **Supplementary Table 2 (Search strategy)**

| **Database** | **Search terms** |
| --- | --- |
| PubMed | 1. ((("Renal Insufficiency, Chronic"[Mesh]) OR (((((((((((((((((((Chronic Renal Insufficiencies[Title/Abstract]) OR (Renal Insufficiencies, Chronic[Title/Abstract])) OR (Chronic Renal Insufficiency[Title/Abstract])) OR (Kidney Insufficiency, Chronic[Title/Abstract])) OR (Chronic Kidney Insufficiency[Title/Abstract])) OR (Chronic Kidney Insufficiencies[Title/Abstract])) OR (Kidney Insufficiencies, Chronic[Title/Abstract])) OR (Chronic Kidney Diseases[Title/Abstract])) OR (Chronic Kidney Disease[Title/Abstract])) OR (Disease, Chronic Kidney[Title/Abstract])) OR (Diseases, Chronic Kidney[Title/Abstract])) OR (Kidney Disease, Chronic[Title/Abstract])) OR (Kidney Diseases, Chronic[Title/Abstract])) OR (Chronic Renal Diseases[Title/Abstract])) OR (Chronic Renal Disease[Title/Abstract])) OR (Disease, Chronic Renal[Title/Abstract])) OR (Diseases, Chronic Renal[Title/Abstract])) OR (Renal Disease, Chronic[Title/Abstract])) OR (Renal Diseases, Chronic[Title/Abstract]))) OR (((((((((((Kidney Failure, Chronic[Title/Abstract]) ) OR (Kidney diseases[Title/Abstract])) OR (Renal Insufficiency[Title/Abstract])) OR (Renal Replacement Therapy[Title/Abstract])) OR (Diabetic Nephropath*[Title/Abstract])) OR (Hypertension, Renal[Title/Abstract])) OR (Hypertension, Renovascular[Title/Abstract])) OR (Azotemia[Title/Abstract])) OR (Hypertensive nephropathy[Title/Abstract])) OR (pre-dialysis[Title/Abstract]))) OR (("Kidney Transplantation"[Mesh]) OR (((((((((((Renal Transplantation[Title/Abstract]) OR (Renal Transplantations[Title/Abstract])) OR (Transplantations, Renal[Title/Abstract])) OR (Transplantation, Renal[Title/Abstract])) OR (Grafting, Kidney[Title/Abstract])) OR (Kidney Grafting[Title/Abstract])) OR (Transplantation, Kidney[Title/Abstract])) OR (Kidney Transplantations[Title/Abstract])) OR (Transplantations, Kidney[Title/Abstract])) OR (kidney transplant recipients[Title/Abstract])) OR (KTRs[Title/Abstract]))) 2. ((Sodium Bicarbonate[MeSH Terms]) OR (((((((((((Bicarbonate, Sodium[Title/Abstract]) OR (Carbonic Acid Monosodium Salt[Title/Abstract])) OR (Sodium Hydrogen Carbonate[Title/Abstract])) OR (Hydrogen Carbonate, Sodium[Title/Abstract])) OR (Baking Soda[Title/Abstract])) OR (Soda, Baking[Title/Abstract])) OR (Oral Sodium Bicarbonate[Title/Abstract])) OR (bicarbonate supplementation[Title/Abstract])) OR (Alkali Therapy[Title/Abstract])) OR (Oral bicarbonate[Title/Abstract])))) 3. ((((randomized controlled trial[Publication Type] OR randomized[Title/Abstract] OR placebo[Title/Abstract]) OR (randomised[Title/Abstract])) OR (treatment group[Title/Abstract])) OR (control group[Title/Abstract])) 4. 1 AND 2 AND 3 |
| Cochrane Library | 1. MeSH descriptor: [Renal Insufficiency, Chronic] OR (Chronic Renal Insufficiencies):ti,ab,kw OR (Renal Insufficiencies, Chronic):ti,ab,kw OR (Chronic Renal Insufficiency):ti,ab,kw OR (Kidney Insufficiency, Chronic):ti,ab,kw OR (Chronic Kidney Insufficiency):ti,ab,kw (Word variations have been searched) or (Chronic Kidney Insufficiencies):ti,ab,kw OR (Kidney Insufficiencies, Chronic):ti,ab,kw OR (Chronic Kidney Diseases):ti,ab,kw OR (Chronic Kidney Disease):ti,ab,kw OR (Disease, Chronic Kidney):ti,ab,kw (Word variations have been searched) or (Diseases, Chronic Kidney):ti,ab,kw OR (Kidney Disease, Chronic):ti,ab,kw OR (Kidney Diseases, Chronic):ti,ab,kw OR (Chronic Renal Diseases):ti,ab,kw OR (Chronic Renal Disease):ti,ab,kw (Word variations have been searched) or (Disease, Chronic Renal):ti,ab,kw OR (Diseases, Chronic Renal):ti,ab,kw OR (Renal Disease, Chronic):ti,ab,kw OR (Renal Diseases, Chronic):ti,ab,kw OR (Kidney Failure, Chronic):ti,ab,kw (Word variations have been searched) or (Kidney diseases):ti,ab,kw OR (Renal Insufficiency):ti,ab,kw OR (Renal Replacement Therapy):ti,ab,kw OR (Diabetic Nephropath*):ti,ab,kw OR (Hypertension, Renal):ti,ab,kw (Word variations have been searched) or (Hypertension, Renovascular):ti,ab,kw OR (Azotemia):ti,ab,kw OR (Hypertensive nephropathy):ti,ab,kw OR (pre-dialysis):ti,ab,kw (Word variations have been searched) 2. MeSH descriptor: [Kidney Transplantation] OR (Renal Transplantation):ti,ab,kw OR (Renal Transplantations):ti,ab,kw OR (Transplantations, Renal):ti,ab,kw OR (Transplantation, Renal):ti,ab,kw OR (Grafting, Kidney):ti,ab,kw (Word variations have been searched) or (Kidney Grafting):ti,ab,kw OR (Transplantation, Kidney):ti,ab,kw OR (Kidney Transplantations):ti,ab,kw OR (Transplantations, Kidney):ti,ab,kw OR (kidney transplant recipients):ti,ab,kw (Word variations have been searched) or (KTRs):ti,ab,kw (Word variations have been searched) 3. 1 OR 2 4. MeSH descriptor: [Sodium Bicarbonate] OR (Bicarbonate, Sodium):ti,ab,kw OR (Carbonic Acid Monosodium Salt):ti,ab,kw OR (Sodium Hydrogen Carbonate):ti,ab,kw OR (Hydrogen Carbonate, Sodium):ti,ab,kw OR (Baking Soda):ti,ab,kw (Word variations have been searched) or (Soda, Baking):ti,ab,kw OR (Oral Sodium Bicarbonate):ti,ab,kw OR (bicarbonate supplementation):ti,ab,kw OR (Alkali Therapy):ti,ab,kw OR (Oral bicarbonate):ti,ab,kw (Word variations have been searched) 5. 3 AND 4 |
| Embase | 1. 'chronic kidney failure'/exp OR 'renal insufficiency, chronic':ab,ti OR 'chronic renal insufficiencies':ab,ti OR 'renal insufficiencies, chronic':ab,ti OR 'chronic renal insufficiency':ab,ti OR 'kidney insufficiency, chronic':ab,ti OR 'chronic kidney insufficiency':ab,ti OR 'chronic kidney diseases':ab,ti OR 'chronic kidney disease':ab,ti OR 'disease, chronic kidney':ab,ti OR 'diseases, chronic kidney':ab,ti OR 'kidney disease, chronic':ab,ti OR 'kidney diseases, chronic':ab,ti OR 'chronic renal diseases':ab,ti OR 'chronic renal disease':ab,ti OR 'disease, chronic renal':ab,ti OR 'diseases, chronic renal':ab,ti OR 'renal disease, chronic':ab,ti OR 'renal diseases, chronic':ab,ti OR 'kidney failure, chronic':ab,ti OR 'kidney diseases':ab,ti OR 'renal insufficiency':ab,ti OR 'renal replacement therapy':ab,ti OR 'diabetic nephropath*':ab,ti OR 'hypertension, renal': ab,ti OR 'hypertension, renovascular':ab,ti OR 'azotemia':ab,ti OR 'hypertensive nephropathy':ab,ti OR 'pre-dialysis':ab,ti OR 'kidney transplantation'/exp OR 'renal transplantation':ab,ti OR 'renal transplantations':ab,ti OR 'transplantations, renal': ab,ti OR 'transplantation, renal':ab,ti OR 'grafting, kidney':ab,ti OR 'kidney grafting':ab,ti OR 'transplantation, kidney':ab,ti OR 'kidney transplantations':ab,ti OR 'transplantations, kidney':ab,ti OR 'kidney transplant recipients':ab,ti OR 'ktrs':ab,ti 2. 'bicarbonate'/exp OR 'sodium bicarbonate':ab,ti OR 'bicarbonate, sodium':ab,ti OR 'carbonic acid monosodium salt':ab,ti OR 'sodium hydrogen carbonate':ab,ti OR 'hydrogen carbonate, sodium':ab,ti OR 'baking soda':ab,ti OR 'soda, baking':ab,ti OR 'oral sodium bicarbonate':ab,ti OR 'bicarbonate supplementation':ab,ti OR 'alkali therapy':ab,ti OR 'oral bicarbonate':ab,ti 3. random:ab,ti OR placebo:ab,ti OR 'double blind':ab,ti 4. 1 AND 2 AND 3 |
| Web of Science | 1. TS=(Renal Insufficienc*, Chronic OR Chronic Renal Insufficienc* OR Kidney Insufficienc*, Chronic OR Chronic Kidney Insufficienc* OR Chronic Kidney Disease* OR Disease*, Chronic Kidney OR Kidney Disease*, Chronic OR Chronic Renal Disease* OR Disease*, Chronic Renal OR Renal Disease*, Chronic OR Kidney Failure, Chronic OR Kidney disease* OR Renal Insufficiency OR Renal Replacement Therapy OR Diabetic Nephropath* OR Hypertension, Renal OR Hypertension, Renovascular OR Azotemia OR Hypertensive nephropathy OR pre-dialysis OR Kidney Transplantation* OR Renal Transplantation* OR Transplantation*, Renal OR Grafting, Kidney OR Kidney Grafting OR Transplantation*, Kidney OR kidney transplant recipients OR KTRs) 2. TS=(Sodium Bicarbonate OR Bicarbonate, Sodium OR Carbonic Acid Monosodium Salt OR Sodium Hydrogen Carbonate OR Hydrogen Carbonate, Sodium OR Baking Soda OR Soda, Baking OR Oral Sodium Bicarbonate OR bicarbonate supplementation OR Alkali Therapy OR Oral bicarbonate) 3. TS=(random* controlled trial OR random* OR placebo OR treatment group OR control group) 4. 1 AND 2 AND 3 |

- 1. **Supplementary Table 3 (Univariate / Multivariate meta-regression analysis of kidney function)**

| **meta-regression analysis** | **_ES** | **Coefficient** | **Std. err.** | **t** | **P>\|t\|** | **[95% conf. interval]** | |
| --- | --- | --- | --- | --- | --- | --- | --- |
| Univariate analysis | Study duration | 0.0302207 | 0.0109112 | 2.77 | 0.020 | 0.005909 | 0.0545324 |
|  | Jadad score | -0.4609921 | 0.1658716 | -2.78 | 0.019 | -0.830577 | -0.0914072 |
|  | Type of control group | -0.5074416 | 0.5595256 | -0.91 | 0.386 | -1.754142 | 0.7392591 |
|  | Year of publication | -0.0169833 | 0.0699586 | -0.24 | 0.813 | -0.1728607 | 0.1388942 |
|  | Male ratio | -0.0259659 | 0.0252435 | -1.03 | 0.328 | -0.0822118 | 0.03028 |
|  | Baseline mean kidney-function level | 0.0116608 | 0.0193495 | 0.60 | 0.560 | -0.0314525 | 0.0547742 |
|  | Baseline mean serum-bicarbonate level | 0.0147763 | 0.0963834 | 0.15 | 0.881 | -0.1999793 | 0.229532 |
| Multivariate analysis | Study duration | 0.0247427 | 0.0111582 | 2.22 | 0.057 | -0.0009882 | 0.0504737 |
|  | Jadad score | -0.2471608 | 0.1861821 | -1.33 | 0.221 | -0.6764976 | 0.1821759 |
|  | Type of control group | -0.3560039 | 0.4432507 | -0.80 | 0.445 | -1.378142 | 0.6661341 |
|  | Year of publication | 0.0935063 | 0.051723 | 1.81 | 0.145 | -0.0500998 | 0.2371123 |
|  | Male ratio | 0.004377 | 0.0234254 | 0.19 | 0.861 | -0.0606623 | 0.0694163 |
|  | Baseline mean kidney-function level | -0.0200982 | 0.0268616 | -0.75 | 0.496 | -0.0946778 | 0.0544815 |
|  | Baseline mean serum-bicarbonate level | -0.099506 | 0.114966 | -0.87 | 0.436 | -0.418703 | 0.2196909 |
|  | _cons | -186.5023 | 103.6797 | -1.80 | 0.146 | -474.3633 | 101.3587 |

**1.4 Supplementary Table 4 (Univariate / Multivariate meta-regression analysis of serum bicarbonate)**

| **meta-regression analysis** | **_ES** | **Coefficient** | **Std. err.** | **t** | **P>\|t\|** | **[95% conf. interval]** | |
| --- | --- | --- | --- | --- | --- | --- | --- |
| Univariate analysis | Study duration | 0 .0048595 | 0.0129392 | 0.38 | 0.714 | -0.0233325 | 0.0330515 |
|  | Jadad score | -0.2854595 | 0.1787398 | -1.60 | 0.136 | -0.6749 | 0.103981 |
|  | Type of control group | 1.204288 | 0.3457199 | 3.48 | 0.005 | 0.4510296 | 1.957547 |
|  | Year of publication | -0.0324895 | 0.1178346 | -0.28 | 0.787 | -0.2892291 | 0.22425 |
|  | Male ratio | 0.0268741 | 0.033086 | 0.81 | 0.432 | -0.045214 | 0.0989623 |
|  | Stage of CKD | 0.78599 | 0.7778872 | 1.01 | 0.332 | -0.9088806 | 2.480861 |
|  | Baseline mean serum-bicarbonate level | -0.3166209 | 0.1410653 | -2.24 | 0.044 | -0.6239758 | -0.009266 |
| Multivariate analysis | Study duration | -0.021497 | 0.0126019 | -1.71 | 0.119 | 0.0495759 | 0.0065818 |
|  | Jadad score | 0.2959987 | 0.2331328 | 1.27 | 0.233 | -0.2234536 | 0.815451 |
|  | Type of control group | 3.114779 | 0.5664014 | 5.50 | 0.000 | 1.852758 | 4.3768 |
|  | Year of publication | -0.0562827 | 0.0788534 | -0.71 | 0.502 | -0.2492299 | 0.1366645 |
|  | Male ratio | 0.0193935 | 0.0243729 | 0.80 | 0.457 | -0.0402447 | 0.0790317 |
|  | Stage of CKD | -1.088262 | 0.8127447 | -1.34 | 0.229 | -3.076977 | 0.9004528 |
|  | Baseline mean serum-bicarbonate level | -0.1305996 | 0.1488175 | -0.88 | 0.414 | -0.494743 | 0.2335438 |
|  | _cons | 122.6613 | 158.0799 | 0.78 | 0.467 | -264.1462 | 509.4688 |

**1.5 Supplementary Table 5 (Sensitivity analysis of kidney function [eGFR or Ccl] in meta-analysis using** **the one-by-one elimination method )**

| **Study ID** | **SMD 95%CI** | **P value** | **I^2^** | **P value** |
| --- | --- | --- | --- | --- |
| Omitting Alva2020 | 0.51 [0.13, 0.89] | 0.009 | 90% | <0.00001 |
| Omitting Bellasi 2016 | 0.52 [0.12, 0.92] | 0.01 | 90% | <0.00001 |
| Omitting Bovée 2021 | 0.53 [0.16, 0.90] | 0.005 | 90% | <0.00001 |
| Omitting de Brito-Ashurst2009 | 0.50 [0.10, 0.89] | 0.01 | 90% | <0.00001 |
| Omitting Dubey 2020 | 0.46 [0.08, 0.85] | 0.02 | 89% | <0.00001 |
| Omitting Goraya 2019 | 0.29 [0.08, 0.50] | 0.007 | 66% | 0.001 |
| Omitting Kendrick 2018 | 0.54 [0.17, 0.91] | 0.004 | 90% | <0.00001 |
| Omitting Kendrick 2023 | 0.54 [0.15, 0.92] | 0.006 | 89% | <0.00001 |
| Omitting Mahajan 2010 | 0.48 [0.10, 0.87] | 0.01 | 90% | <0.00001 |
| Omitting Melamed 2020 | 0.54 [0.16, 0.93] | 0.006 | 89% | <0.00001 |
| Omitting Raphael 2020B | 0.55 [0.18, 0.93] | 0.004 | 89% | <0.00001 |
| Omitting Yan 2017 | 0.49 [0.10, 0.87] | 0.01 | 90% | <0.00001 |

**1.6 Supplementary Table 6 (Sensitivity analysis of serum bicarbonate in meta-analysis using the one-by-one elimination method)**

| **Study ID** | **SMD 95%CI** | **P value** | **I^2^** | **P value** |
| --- | --- | --- | --- | --- |
| Omitting Alva2020 | 2.24 [1.25, 3.23] | <0.00001 | 96% | <0.00001 |
| Omitting Bellasi 2016 | 2.18 [1.23, 3.31] | <0.00001 | 95% | <0.00001 |
| Omitting Bovée 2021 | 2.28 [1.29, 3.28] | <0.00001 | 96% | <0.00001 |
| Omitting de Brito-Ashurst2009 | 2.29 [1.32, 3.26] | <0.00001 | 96% | <0.00001 |
| Omitting Dubey 2020 | 1.99 [1.19, 2.79] | <0.00001 | 93% | <0.00001 |
| Omitting Goraya 2019 | 2.43 [1.23, 3.63] | <0.0001 | 96% | <0.00001 |
| Omitting Kendrick 2018 | 2.32 [1.33, 3.32] | <0.00001 | 96% | <0.00001 |
| Omitting Kendrick 2023 | 2.44 [1.42, 3.46] | <0.00001 | 96% | <0.00001 |
| Omitting Mahajan 2010 | 2.54 [1.60, 3.48] | <0.00001 | 94% | <0.00001 |
| Omitting Mathur 2006 | 2.24 [1.27, 3.21] | <0.00001 | 96% | <0.00001 |
| Omitting Melamed 2020 | 2.48 [1.46, 3.50] | <0.00001 | 96% | <0.00001 |
| Omitting Raphael 2020A | 2.45 [1.44, 3.46] | <0.00001 | 96% | <0.00001 |
| Omitting Raphael 2020B | 2.48 [1.46, 3.50] | <0.00001 | 96% | <0.00001 |
| Omitting Yan 2017 | 2.51 [1.48, 3.53] | <0.00001 | 96% | <0.00001 |

**1.7 Supplementary Table 7 (Sensitivity analysis of blood pH in meta-analysis using the one-by-one elimination method)**

| **Study ID** | **SMD 95%CI** | **P value** | **I^2^** | **P value** |
| --- | --- | --- | --- | --- |
| Omitting Dubey 2020 | 0.77 [-0.05, 0.19] | 0.25 | 97% | <0.00001 |
| Omitting Kendrick 2023 | 0.17 [0.10, 0.24] | <0.00001 | 92% | 0.0003 |
| Omitting Mathur 2006 | 0.11 [-0.08, 0.29] | 0.27 | 100% | <0.00001 |

**1.8 Supplementary Table 8 (Sensitivity analysis of systolic blood pressure in meta-analysis using the one-by-one elimination method)**

| **Study ID** | **SMD 95%CI** | **P value** | **I^2^** | **P value** |
| --- | --- | --- | --- | --- |
| Omitting Bellasi 2016 | -0.15 [-1.77, 1.47] | 0.86 | 0% | 0.69 |
| Omitting Bovée 2021 | -0.12 [-1.70, 1.45] | 0.88 | 0% | 0.70 |
| Omitting de Brito-Ashurst2009 | -0.09 [-1.87, 1.69] | 0.92 | 0% | 0.70 |
| Omitting Dubey 2020 | 0.08 [-1.54, 1.70] | 0.92 | 0% | 0.79 |
| Omitting Goraya 2019 | 0.24 [-1.38, 1.86] | 0.77 | 0% | 0.95 |
| Omitting Kendrick 2018 | -0.11 [-1.69, 1.47] | 0.89 | 0% | 0.70 |
| Omitting Kendrick 2023 | -0.24 [-1.86, 1.38] | 0.77 | 0% | 0.72 |
| Omitting Mahajan 2010 | -0.34 [-1.99, 1.31] | 0.69 | 0% | 0.75 |
| Omitting Mathur 2006 | -0.66 [-2.40, 1.08] | 0.46 | 0% | 0.86 |
| Omitting Melamed 2020 | 0.01 [-1.62, 1.63] | 0.99 | 0% | 0.74 |
| Omitting Raphael 2020B | -0.21 [-1.86, 1.44] | 0.80 | 0% | 0.70 |

**1.9 Supplementary Table 9 (Sensitivity analysis of diastolic blood pressure in meta-analysis using the one-by-one elimination method)**

| **Study ID** | **SMD 95%CI** | **P value** | **I^2^** | **P value** |
| --- | --- | --- | --- | --- |
| Omitting Bellasi 2016 | 1.80 [0.08, 3.51] | 0.04 | 20% | 0.27 |
| Omitting Bovée 2021 | 2.15 [0.47, 3.84] | 0.01 | 33% | 0.18 |
| Omitting de Brito-Ashurst2009 | 2.23 [0.47, 4.00] | 0.01 | 32% | 0.19 |
| Omitting Dubey 2020 | 2.99 [1.57, 4.41] | <0.0001 | 0% | 0.63 |
| Omitting Kendrick 2018 | 2.43 [0.93, 3.92] | 0.001 | 17% | 0.30 |
| Omitting Kendrick 2023 | 2.26 [0.52, 4.01] | 0.01 | 31% | 0.19 |
| Omitting Mathur 2006 | 1.71 [0.05, 3.37] | 0.04 | 11% | 0.35 |
| Omitting Melamed 2020 | 1.98 [0.15, 3.81] | 0.03 | 32% | 0.18 |

**1.10 Supplementary Table 10 (Sensitivity analysis of** **AEs in meta-analysis using the one-by-one elimination method)**

| **Study ID** | **SMD 95%CI** | **P value** | **I^2^** | **P value** |
| --- | --- | --- | --- | --- |
| Omitting Dubey 2020 | 1.07 [0.73, 1.58] | 0.71 | 73% | 0.005 |
| Omitting Kendrick 2018 | 1.21 [0.81, 1.80] | 0.35 | 86% | <0.00001 |
| Omitting Kendrick 2023 | 1.38 [0.77, 2.47] | 0.28 | 89% | <0.00001 |
| Omitting Mathur 2006 | 1.23 [0.81, 1.88] | 0.33 | 88% | <0.00001 |
| Omitting Melamed 2020 | 1.60 [0.92, 2.77] | 0.10 | 88% | <0.00001 |
| Omitting Raphael 2020A | 1.30 [0.84, 2.00] | 0.24 | 86% | <0.00001 |
| Omitting Raphael 2020B | 1.51 [0.82, 2.77] | 0.18 | 85% | <0.0001 |

**1.11 Supplementary Table 11 (Sensitivity analysis of edema in meta-analysis using the one-by-one elimination method)**

| **Study ID** | **SMD 95%CI** | **P value** | **I^2^** | **P value** |
| --- | --- | --- | --- | --- |
| Omitting de Brito-Ashurst2009 | 1.27 [0.96, 1.69] | 0.10 | 46% | 0.10 |
| Omitting Dubey 2020 | 1.15 [0.88, 1.51] | 0.31 | 27% | 0.23 |
| Omitting Kendrick 2018 | 1.23 [0.96, 1.57] | 0.10 | 31% | 0.20 |
| Omitting Kendrick 2023 | 1.28 [1.00, 1.64] | 0.05 | 46% | 0.10 |
| Omitting Mathur 2006 | 1.25 [0.98, 1.60] | 0.07 | 40% | 0.14 |
| Omitting Melamed 2020 | 1.51 [1.07, 2.12] | 0.02 | 26% | 0.24 |
| Omitting Raphael 2020B | 1.34 [1.05, 1.72] | 0.02 | 19% | 0.29 |

**1.12 Supplementary Table 12 (Sensitivity analysis of gastrointestinal disorders in meta-analysis using the one-by-one elimination method)**

| **Study ID** | **SMD 95%CI** | **P value** | **I^2^** | **P value** |
| --- | --- | --- | --- | --- |
| Omitting Dubey 2020 | 1.04 [0.90, 1.20] | 0.60 | 0% | 0.50 |
| Omitting Kendrick 2018 | 1.67 [0.62, 4.48] | 0.31 | 74% | 0.01 |
| Omitting Kendrick 2023 | 1.84 [0.61, 5.57] | 0.28 | 75% | 0.008 |
| Omitting Mahajan 2010 | 1.13 [0.65, 1.96] | 0.66 | 56% | 0.08 |
| Omitting Raphael 2020B | 1.91 [0.59, 6.13] | 0.28 | 66% | 0.03 |

**1.13 Supplementary Table 13 (Sensitivity analysis of worsening hypertension in meta-analysis using the one-by-one elimination method)**

| **Study ID** | **SMD 95%CI** | **P value** | **I^2^** | **P value** |
| --- | --- | --- | --- | --- |
| Omitting de Brito-Ashurst2009 | 1.68 [1.07, 2.64] | 0.03 | 0% | 0.59 |
| Omitting Dubey 2020 | 1.36 [0.99, 1.87] | 0.05 | 0% | 0.50 |
| Omitting Mathur 2006 | 1.39 [1.07, 1.82] | 0.02 | 0% | 0.76 |
| Omitting Raphael 2020B | 1.45 [1.11, 1.89] | 0.007 | 0% | 0.40 |

**1.14 Supplementary Table 14 (Egger's test of change in outcomes)**

| **Outcomes** | **_ES** | **Coefficient** | **Std. err.** | **t** | **P>\|t\|** | **[95% conf. interval]** | |
| --- | --- | --- | --- | --- | --- | --- | --- |
| Kidney function | slope | -0.331613 | 0.6332633 | -0.52 | 0.612 | -1.742612 | 1.079386 |
|  | bias | 3.641099 | 3.105642 | 1.17 | 0.268 | -3.278704 | 10.5609 |
| Serum bicarbonate | slope | 1.012595 | 0.7393517 | 1.37 | 0.196 | -0.5983138 | 2.623504 |
|  | bias | 2.528339 | 2.230027 | 1.13 | 0.279 | -2.330472 | 7.38715 |
| Systolic blood | slope | 2.043399 | 1.873508 | 1.09 | 0.304 | -2.194771 | 6.28157 |
| pressure | bias | -0.8742821 | 0.7078114 | -1.24 | 0.248 | -2.475463 | 0.7268986 |

**1.15 Supplementary Table 15 (Summary of findings of GRADE for the outcomes)**

| Outcomes | Participants(studies)  Follow-up | Overall certainty of evidence | Relative effect(95% CI) | Anticipated absolute effects | |
| --- | --- | --- | --- | --- | --- |
|  |  |  |  | Risk with Control | Risk difference with Sodium Bicarbonate |
| Kidney function (Kidney transplant recipients) | 280 (2 RCTs) | ⨁⨁◯◯ Low^a,b^ | — | — | SMD **0.07 lower**  (0.3 lower to 0.16 higher) |
| Kidney function (Non-transplant patients with CKD) | 1269 (12 RCTs) | ⨁◯◯◯ Very low^a,b,c^ | — | — | SMD **0.49 higher**  (0.14 higher to 0.85 higher) |
| Serum bicarbonate (Kidney transplant recipients) | 280 (2 RCTs) | ⨁◯◯◯ Very low^a,b,c^ | — | The mean serum bicarbonate was **0** | MD **0.76 higher**  (0.38 lower to 1.9 higher) |
| Serum bicarbonate(Non-transplant patients with CKD) | 1380 (14 RCTs) | ⨁◯◯◯ Very low^a,b,c^ | — | The mean serum bicarbonate was **0** | MD **2.35 higher**  (1.4 higher to 3.3 higher) |
| Blood pH (Kidney transplant recipients) | 280 (2 RCTs) | ⨁⨁◯◯ Low^a,d^ | — | The mean blood pH was **0** | MD **0.02 higher**  (0 to 0.04 higher) |
| Blood pH (Non-transplant patients with CKD) | 318 (3 RCTs) | ⨁◯◯◯ Very low^a,d^ | — | The mean blood pH was **0** | MD **0.11 higher**  (0.02 lower to 0.24 higher) |
| Systolic blood pressure (Kidney transplant recipients) | 276 (2 RCTs) | ⨁◯◯◯ Very low^a,b^ | — | The mean systolic blood pressure was **0** | MD **1.57 lower**  (5.2 lower to 2.07 higher) |
| Systolic blood pressure (Non-transplant patients with CKD) | 1158 (11 RCTs) | ⨁⨁◯◯ Low^a,b^ | — | The mean systolic blood pressure was **0** | MD **0.14 lower**  (1.7 lower to 1.43 higher) |
| Diastolic blood pressure (Kidney transplant recipients) | 276 (2 RCTs) | ⨁◯◯◯ Very low^a,b,c^ | — | The mean diastolic blood pressure was **0** | MD **2.41 lower**  (9.03 lower to 4.21 higher) |
| Diastolic blood pressure (Non-transplant patients with CKD) | 812 (8 RCTs) | ⨁⨁◯◯ Low^a,b^ | — | The mean diastolic blood pressure was **0** | MD **2.21 higher**  (0.67 higher to 3.75 higher) |
| Adverse events (Kidney transplant recipients) | 277 (2 RCTs) | ⨁◯◯◯ Very low^a,b,c^ | **RR 0.89**  (0.47 to 1.67) | 300 per 1,000 | **33 fewer per 1,000**  (from 159 fewer to 201 more) |
| Adverse events (Non-transplant patients with CKD) | 794(7 RCTs) | ⨁◯◯◯ Very low^a,b,c^ | **RR 1.30**  (0.84 to 2.00) | 415 per 1,000 | **125 more per 1,000**  (from 66 fewer to 415 more) |
| Edema (Kidney transplant recipients) | 277 (2 RCTs) | ⨁⨁◯◯ Low^a,b^ | **RR 0.76**  (0.33 to 1.75) | 86 per 1,000 | **21 fewer per 1,000**  (from 57 fewer to 64 more) |
| Edema (Non-transplant patients with CKD) | 854 (7 RCTs) | ⨁⨁◯◯ Low^a,b^ | **RR 1.28**  (1.00 to 1.63) | 191 per 1,000 | **54 more per 1,000**  (from 0 fewer to 120 more) |
| Heart failure (Kidney transplant recipients) | 277 (2 RCTs) | ⨁◯◯◯ Very low^a,b^ | **RR 1.03**  (0.06 to 16.21) | 7 per 1,000 | **0 fewer per 1,000**  (from 7 fewer to 109 more) |
| Heart failure (Non-transplant patients with CKD)  Worsening hypertension (Kidney transplant recipients)  Worsening hypertension (Non-renal transplant patients) | 814 (6 RCTs)  277  (2 RCTs)  596  (5 RCTs) | ⨁◯◯◯ Very low^a,b^  ⨁⨁◯◯Low^a,b^  ⨁⨁◯◯Low^a,b^ | **RR 1.81**  (0.40 to 8.22)  **RR 0.79** (0.36 to 1.73)  **RR 1.44** (1.11 to 1.88) | 6 per 1,000  93 per 1,000  209 per 1,000 | **4 more per 1,000**  (from 3 fewer to 40 more)  **19 fewer per 1,000** (from 59 fewer to 68 more)  **92 more per 1,000** (from 23 more to 184 more) |
| Gastrointestinal disorders (Kidney transplant recipients) | 277 (2 RCTs) | ⨁◯◯◯ Very low^a,b,c^ | **RR 0.77**  (0.24 to 2.52) | 136 per 1,000 | **31 fewer per 1,000**  (from 103 fewer to 206 more) |
| Gastrointestinal disorders (Non-transplant patients with CKD) | 725 (6 RCTs) | ⨁◯◯◯ Very low^a,b,c^ | **RR 1.29**  (0.67 to 2.46) | 188 per 1,000 | **55 more per 1,000**  (from 62 fewer to 275 more) |

**CI:** confidence interval; **MD:** mean difference; **RR:** risk ratio; **SMD:** standardised mean difference

#### Explanations

a. Unclear or high risk of bias

b. Wide confidence interval

c. High heterogeneity

d. Including invalid value

# 2 Supplementary Figures

**2.1 Supplementary Figure 1 (Subgroup analysis results of** **the effect of sodium bicarbonate on serum bicarbonate according to the type of control group [placebo, or standard treatment])**


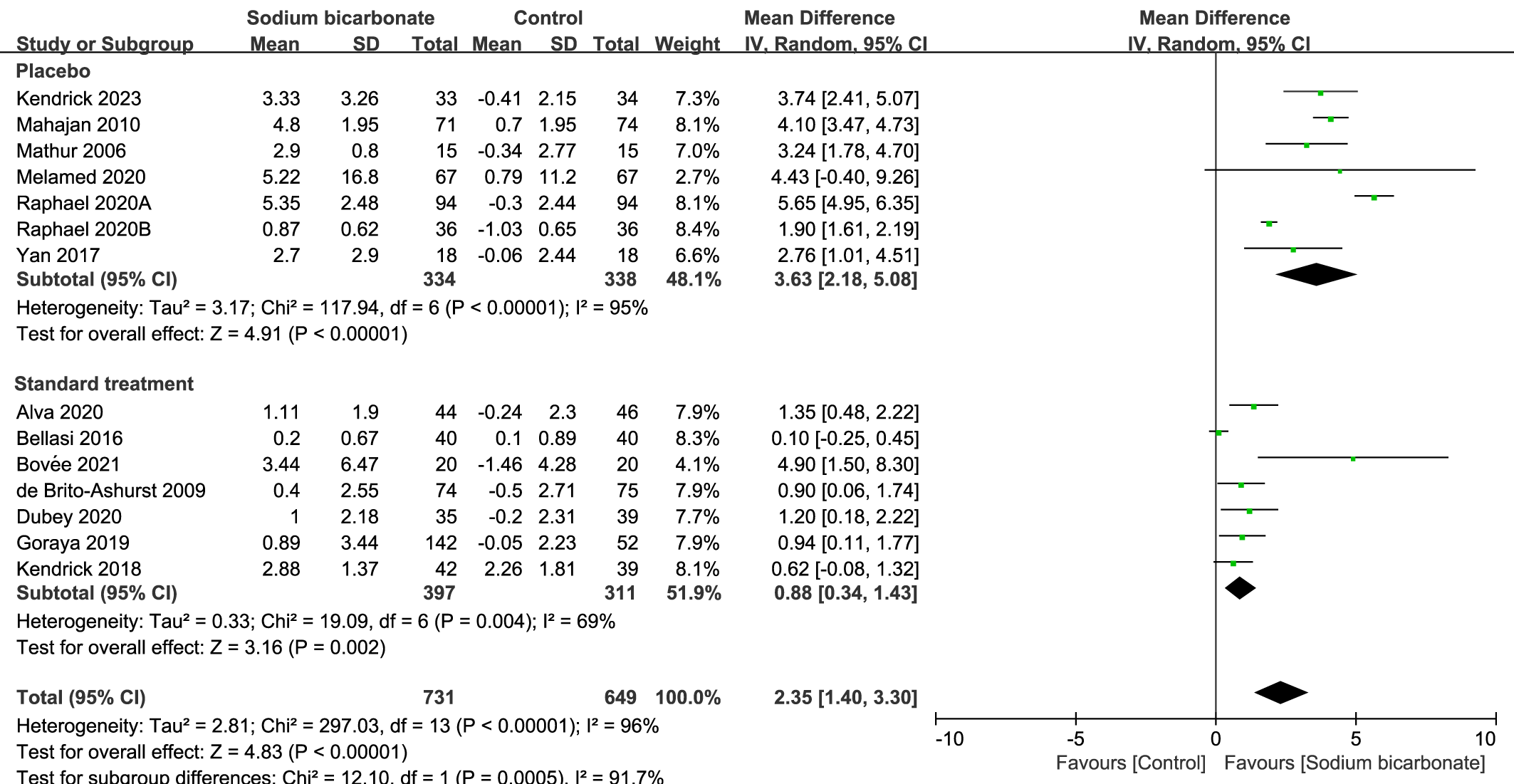


**2.2 Supplementary Figure 2** **(Subgroup analysis results of the effect of sodium bicarbonate on diastolic blood pressure according to the baseline mean eGFR or Ccl level)**


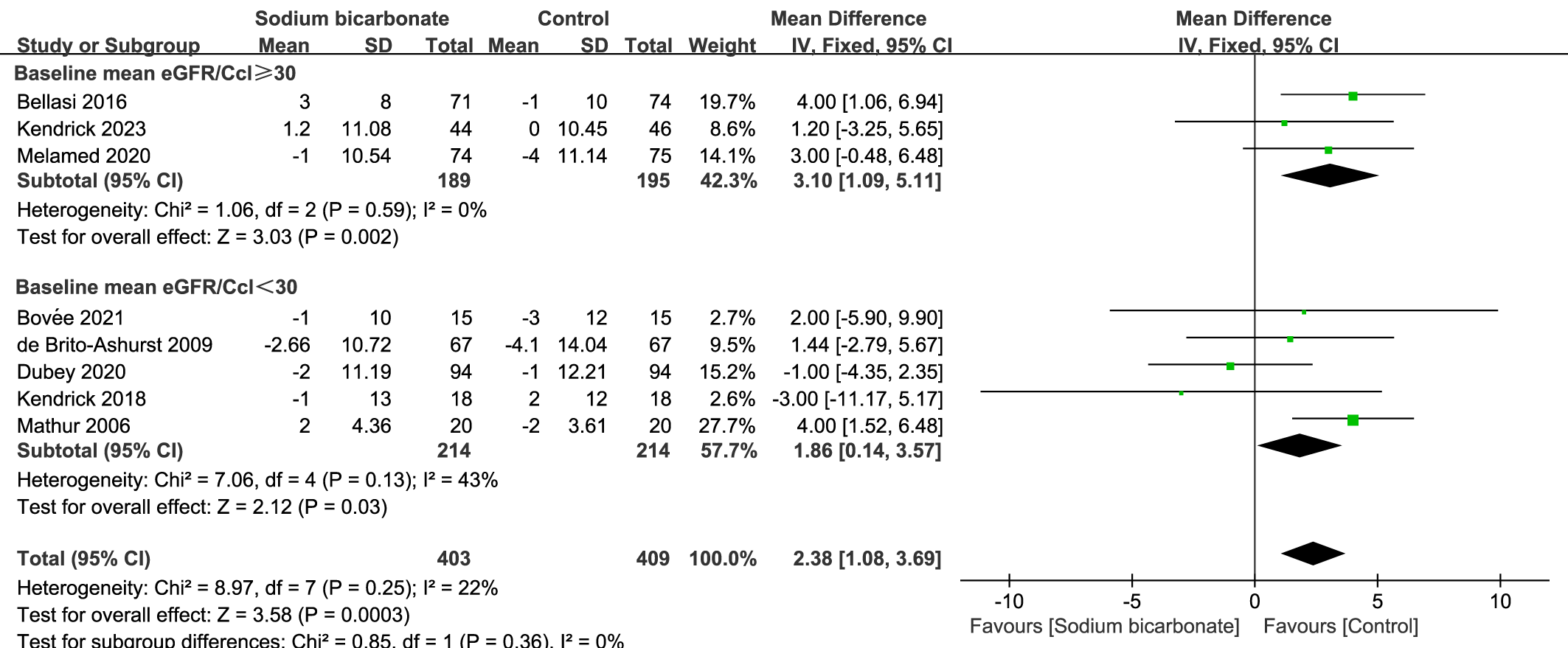


**2.3** **Supplementary Figure 3 (Subgroup analysis results of the effect of sodium bicarbonate on the incidence of edema according to the baseline mean eGFR or Ccl level)**


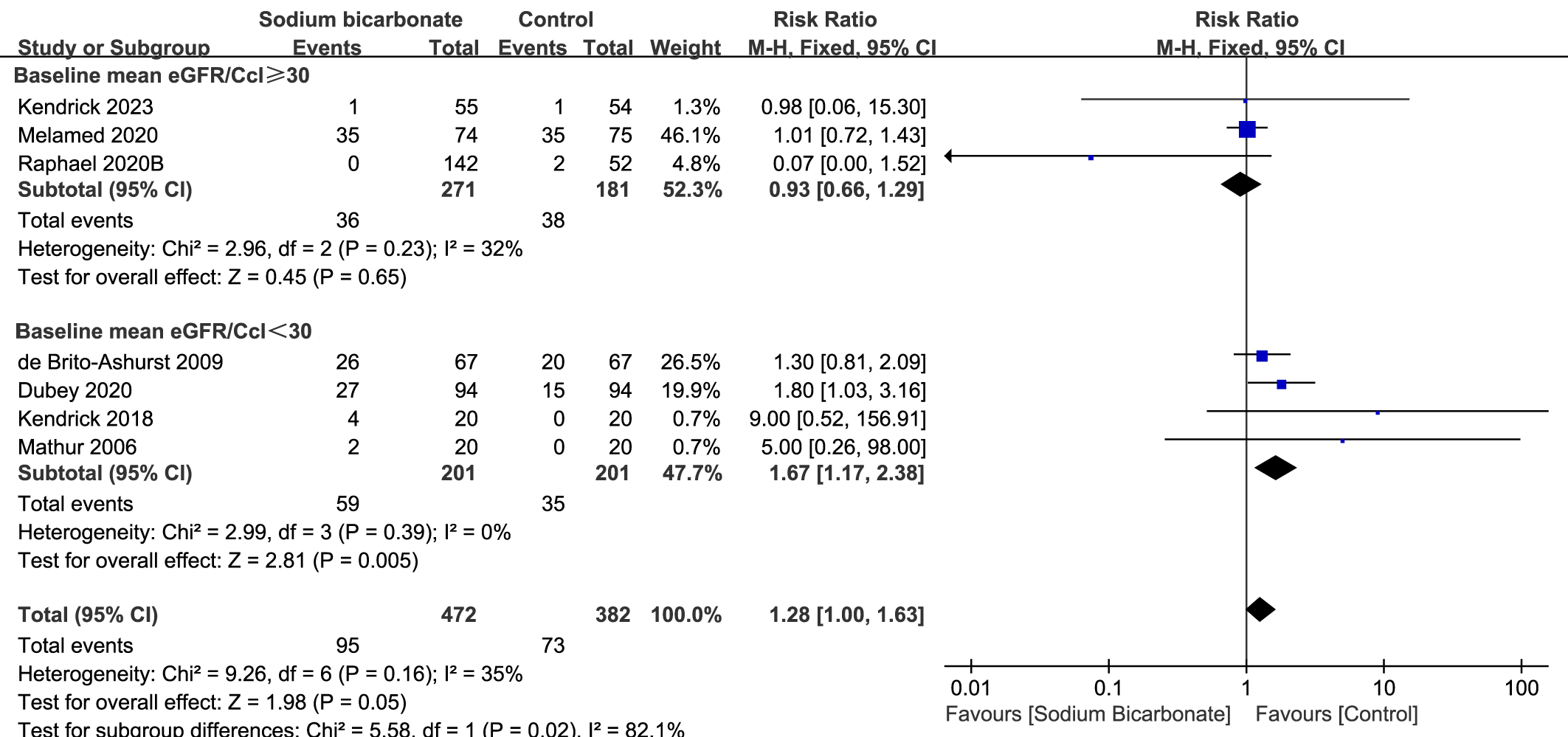


**2.4 Supplementary Figure 4 (Funnel plot of sodium bicarbonate for kidney function)**


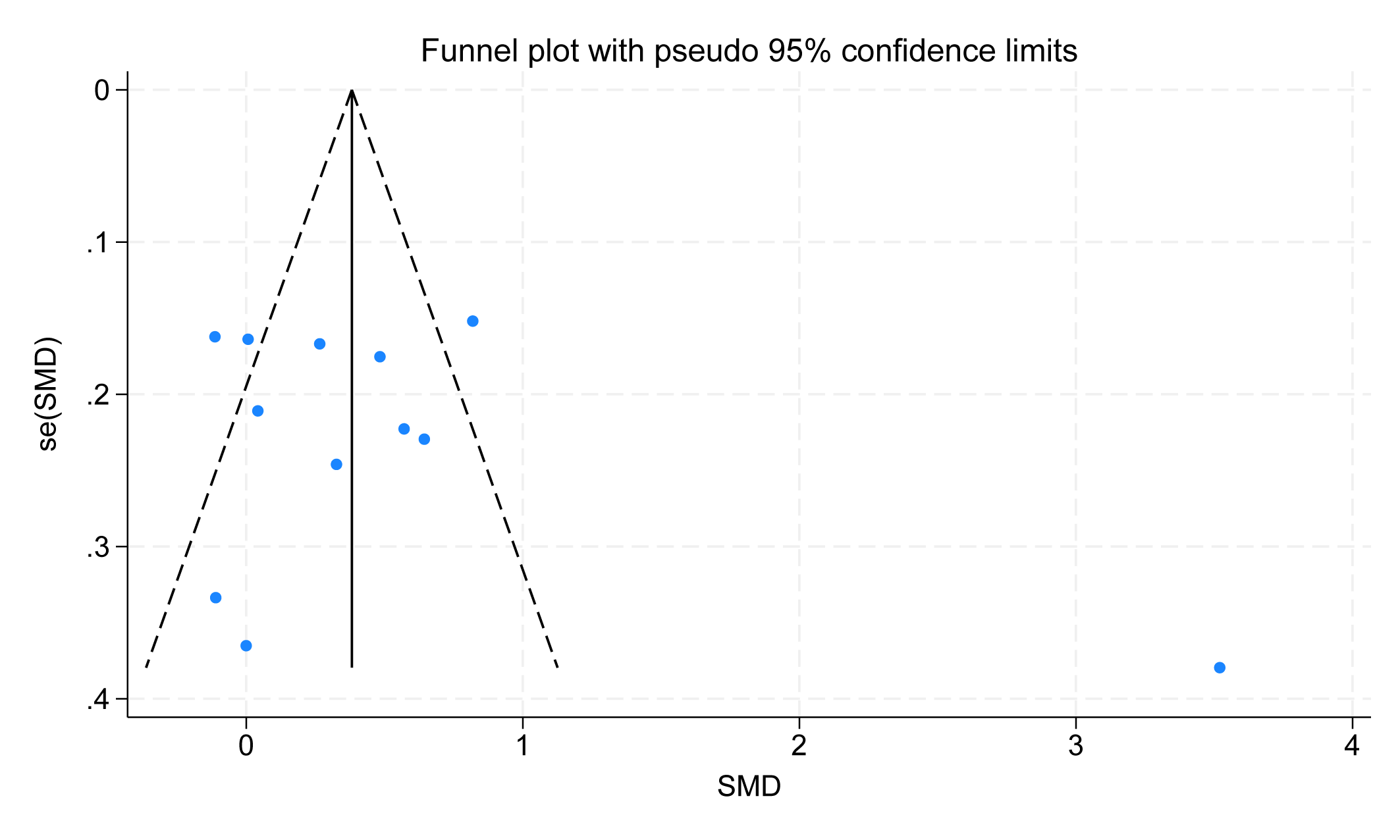


**2.5 Supplementary Figure 5 (Egger’s publication bias plot of sodium bicarbonate for kidney function)**


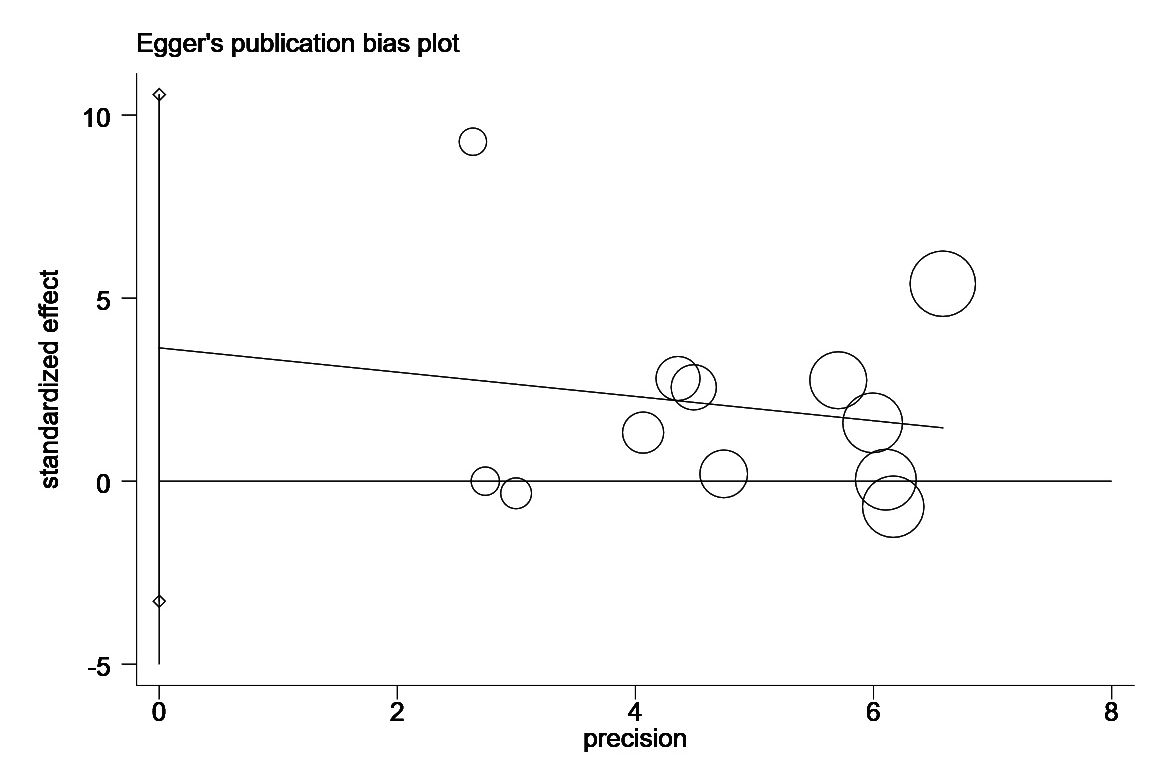


**2.6 Supplementary Figure 6 (Funnel plot of sodium bicarbonate for serum bicarbonate)**


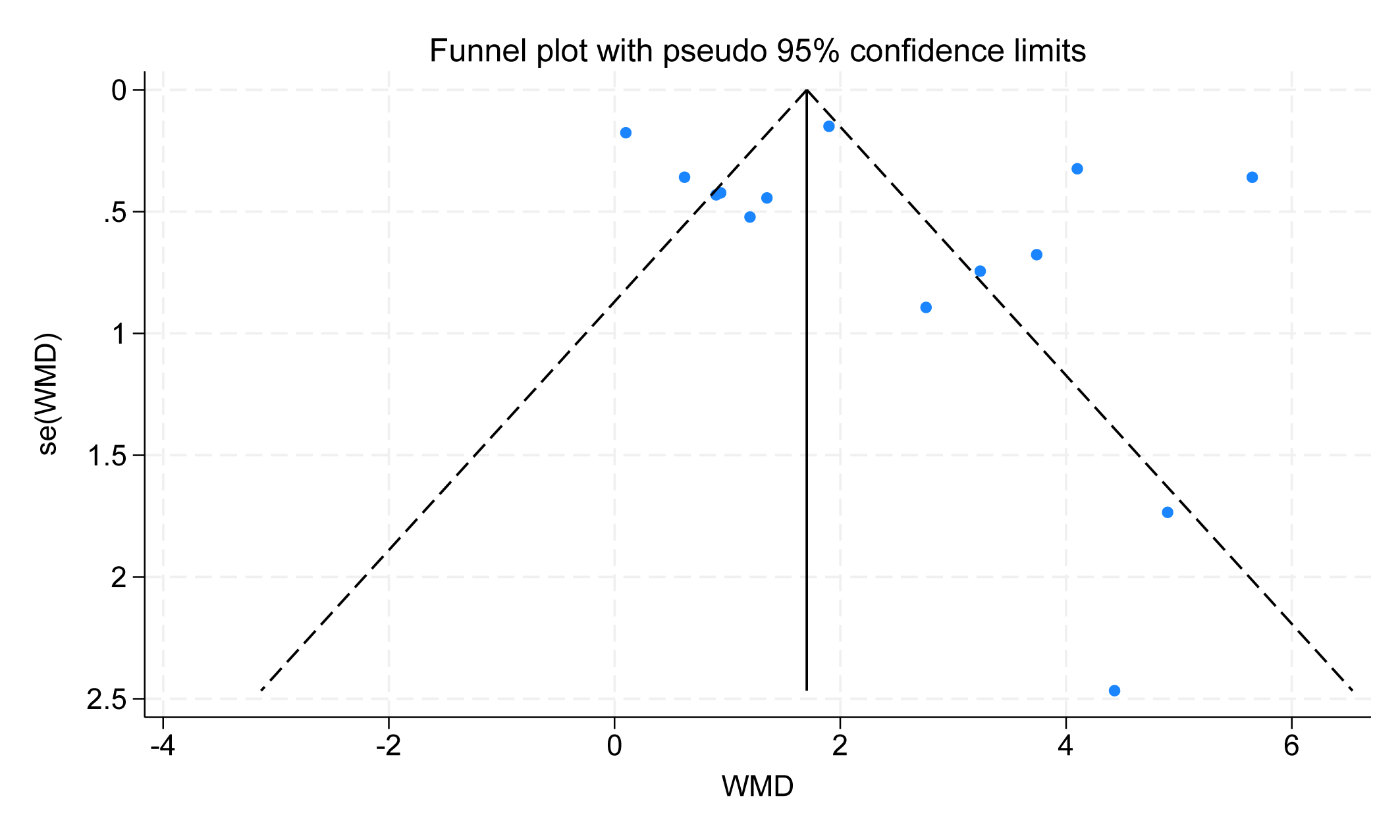


**2.7 Supplementary Figure 7 (Egger’s publication bias plot of sodium bicarbonate for serum bicarbonate)**


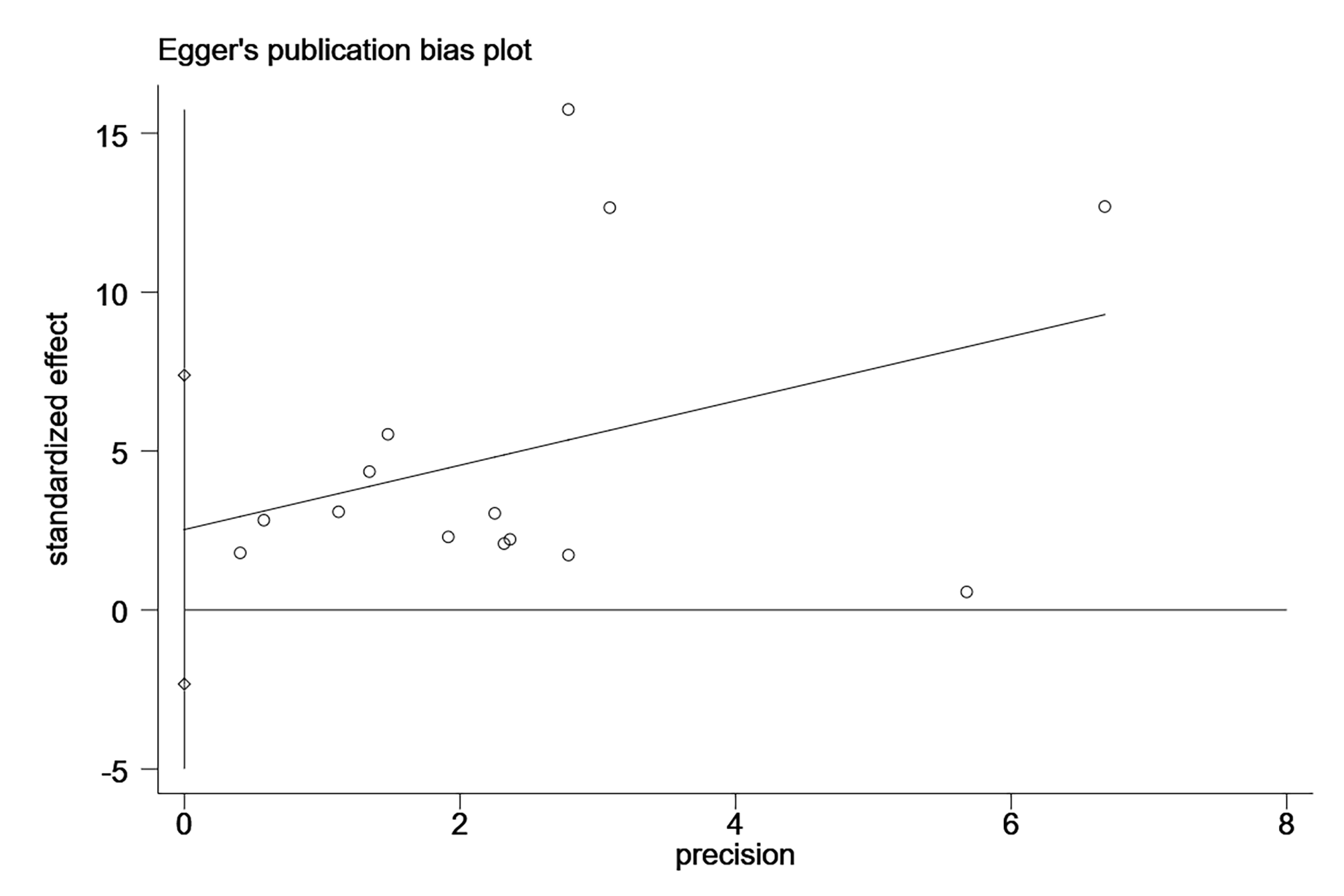


**2.8 Supplementary Figure 8 (Funnel plot of sodium bicarbonate for systolic blood pressure)**


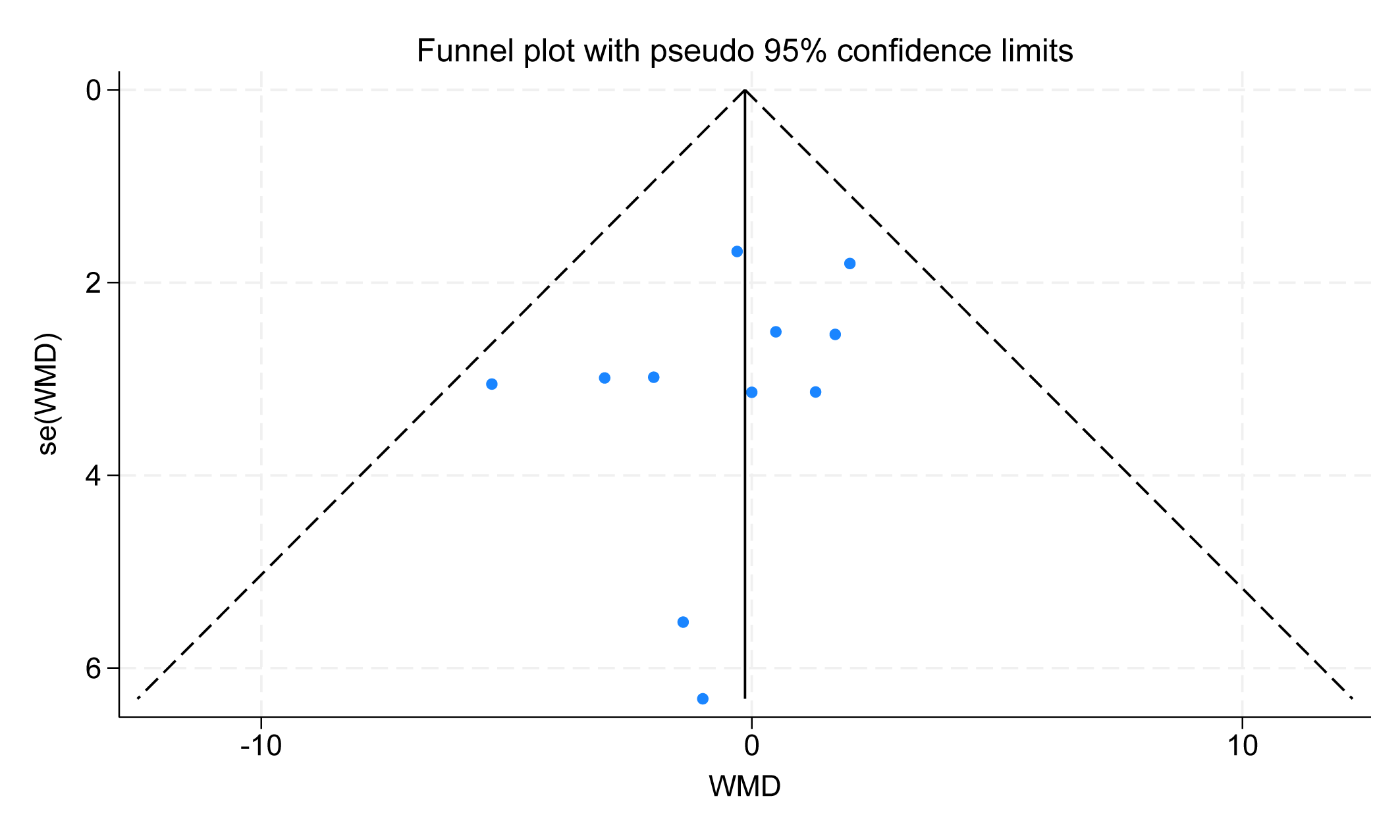


**2.9 Supplementary Figure 9 (Egger’s publication bias plot of sodium bicarbonate for systolic blood pressure)**


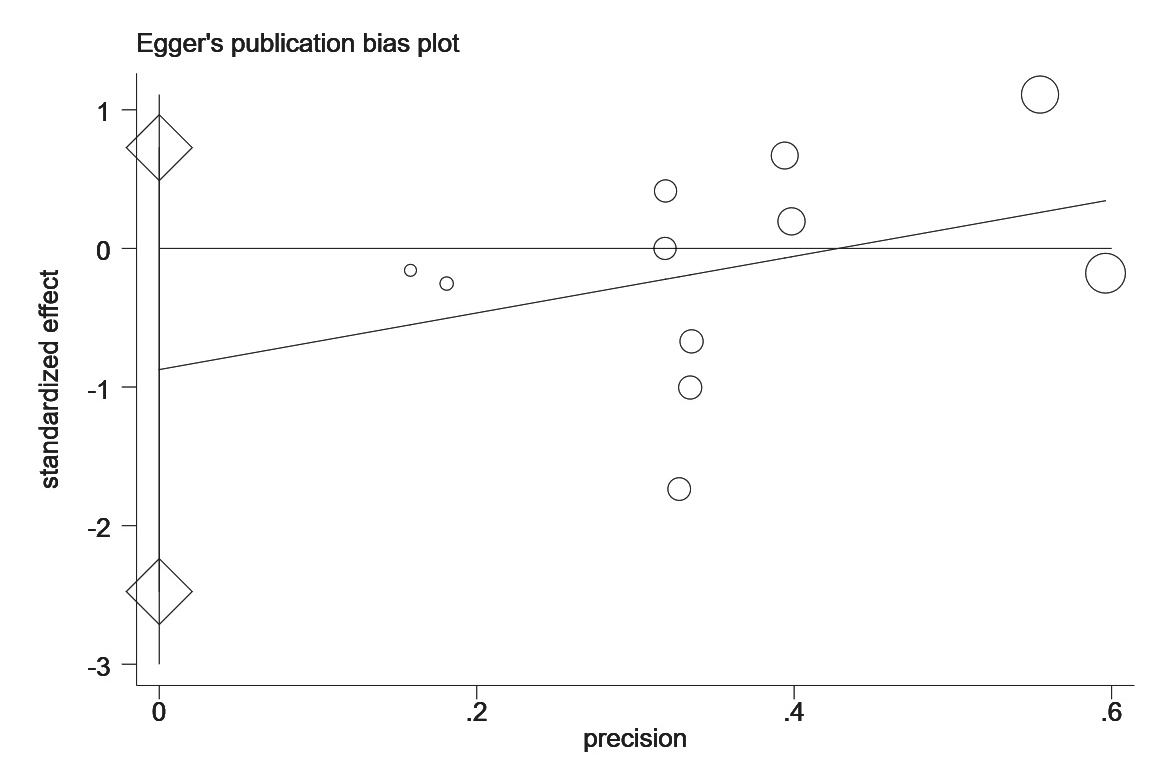

Supplement: Supplementary file 1 [file DataSheet1.docx]
